# SITC cancer immunotherapy resource document: a compass in the land of biomarker discovery

**DOI:** 10.1136/jitc-2020-000705

**Published:** 2020-12-02

**Authors:** Siwen Hu-Lieskovan, Srabani Bhaumik, Kavita Dhodapkar, Jean-Charles J B Grivel, Sumati Gupta, Brent A Hanks, Sylvia Janetzki, Thomas O Kleen, Yoshinobu Koguchi, Amanda W Lund, Cristina Maccalli, Yolanda D Mahnke, Ruslan D Novosiadly, Senthamil R Selvan, Tasha Sims, Yingdong Zhao, Holden T Maecker

**Affiliations:** 1 Huntsman Cancer Institute, Salt Lake City, UT, USA; 2 University of Utah School of Medicine, Salt Lake City, UT, USA; 3 Roche Tissue Diagnostics, Tucson, Arizona, USA; 4 Department of Pediatrics, Emory University, Atlanta, Georgia, USA; 5 Aflac Cancer and Blood Disorders Center, Children's Healthcare of Atlanta, Atlanta, Georgia, USA; 6 Research Department, Sidra Medicine, Doha, Qatar; 7 Huntsman Cancer Institute, Salt Lake City, Utah, USA; 8 Duke University Medical Center, Durham, North Carolina, USA; 9 ZellNet Consulting Inc, Fort Lee, New Jersey, USA; 10 Immodulon Therapeutics Ltd, Uxbridge, UK; 11 Earle A Chiles Research Institute, Providence Cancer Institute, Portland, Oregon, USA; 12 Oregon Health and Science University, Portland, Oregon, USA; 13 FlowKnowHow LLC, Brooklyn, New York, USA; 14 Bristol-Myers Squibb, Princeton, New Jersey, USA; 15 BioMarker Strategies LLC, Rockville, Maryland, USA; 16 Regeneron Pharmaceuticals Inc, Tarrytown, New York, USA; 17 National Cancer Institute, Bethesda, Maryland, USA; 18 Stanford University Medical Center, Stanford, California, USA

**Keywords:** immunotherapy, biomarkers, tumor, antigens, computational biology, immunomodulation

## Abstract

Since the publication of the Society for Immunotherapy of Cancer’s (SITC) original cancer immunotherapy biomarkers resource document, there have been remarkable breakthroughs in cancer immunotherapy, in particular the development and approval of immune checkpoint inhibitors, engineered cellular therapies, and tumor vaccines to unleash antitumor immune activity. The most notable feature of these breakthroughs is the achievement of durable clinical responses in some patients, enabling long-term survival. These durable responses have been noted in tumor types that were not previously considered immunotherapy-sensitive, suggesting that all patients with cancer may have the potential to benefit from immunotherapy. However, a persistent challenge in the field is the fact that only a minority of patients respond to immunotherapy, especially those therapies that rely on endogenous immune activation such as checkpoint inhibitors and vaccination due to the complex and heterogeneous immune escape mechanisms which can develop in each patient. Therefore, the development of robust biomarkers for each immunotherapy strategy, enabling rational patient selection and the design of precise combination therapies, is key for the continued success and improvement of immunotherapy. In this document, we summarize and update established biomarkers, guidelines, and regulatory considerations for clinical immune biomarker development, discuss well-known and novel technologies for biomarker discovery and validation, and provide tools and resources that can be used by the biomarker research community to facilitate the continued development of immuno-oncology and aid in the goal of durable responses in all patients.

## Overview

In the **Introduction to biomarkers for the immunotherapy of cancer** section, we introduce the cancer immunotherapy revolution from the standpoint of biomarkers and their roles in predicting clinical outcome or adverse events, as well as in quantifying antitumor immune responses. We discuss best practices for biomarker development, validation, and harmonization of data, and technical considerations for sample collection and reporting of data. Finally, we review recent biomarker discovery literature and regulatory considerations for developing diagnostics. These topics are divided into the following elements:

- Background.
- Recently approved cancer immunotherapies—a breakthrough.
- Biomarkers of immune response and clinical outcome in patients with cancer.
- Biomarkers of immune-related adverse events and correlation with clinical response.
- Quantifying the antitumor immune response.
- The development and validation of immunotherapy biomarkers.
- Data harmonization efforts for biomarker discovery.
- Sample collection: technical considerations for processing, storage, and shipment of tumor samples for immunological studies.
- Reporting of biomarker data in clinical trials and publications.
- Novel biomarker discovery: immunotherapy biomarker useful literature review.
- Regulatory agency guidelines for diagnostics.

In the **New and emerging technologies for biomarker discovery** section, we focus on technology platforms, especially those that are new and emerging, for use in biomarker discovery. These are grouped by the type of cellular target. First, we consider nucleic acid-based platforms, including genomic, microbiome, mitochondrial genome, epigenetic, transcriptomic (including single-cell), and PCR/hybridization techniques. Second, proteomic biomarkers, ranging from ELISA to mass cytometry, are reviewed, along with imaging technologies that can analyze spatial context in tumor biopsies. Software tools for all these platforms are reviewed. Finally, we review in vivo imaging platforms and metabolic biomarkers. These topics are divided into the following elements:

- Genomic biomarker discovery.
- Microbiome sequencing.
- Mitochondrial genome arrays.
- Epigenomic biomarker discovery.
- Transcriptomic biomarker discovery.
- Single-cell gene expression analysis.
- Hybridization and PCR-based gene expression platforms.
- Proteomic biomarkers discovery: detection techniques.
- Proteomic biomarkers discovery: target identification and immunomonitoring.
- Immune contexture biomarker discovery.
- Software and tools for data analysis.
- In vivo imaging (non-invasive and whole body).
- Predictive metabolic biomarkers in tumor immunotherapy.

We conclude with an extensive table of online resources, including links to consortia and regulatory agency websites, databases, online software tools, and clinical trial registries. Resources include:

- Cancer Immune Monitoring and Analysis Centers/Cancer Immunologic Data Commons network (CIMAC/CIDC).
- Partnership for Accelerating Cancer Therapies (PACT).
- Links to Food and Drug Administration (FDA) biomarker approval.
- Public databases.
- Transcription factors binding sites prediction software.
- Tools for neoantigen prediction.
- Clinical trial registries (CTRs).

## Introduction to biomarkers for the immunotherapy of cancer

### Background

The Society for Immunotherapy of Cancer (SITC) has extensively documented the importance of biomarkers for cancer immunotherapy through symposia and workshops, and the SITC Biomarkers Committee has been involved in the publication of a number of technology primers and white papers.

#### Workshop reports

- SITC 2018 workshop report: immuno-oncology biomarkers: state of the art.^1^
- Immunotherapy biomarkers 2016: overcoming the barriers.^2^

#### White papers

- Validation of biomarkers to predict response to immunotherapy in cancer: volume I—pre-analytical and analytical validation.^3^
- Validation of biomarkers to predict response to immunotherapy in cancer: volume II—clinical validation and regulatory considerations.^4^
- Identifying baseline immune-related biomarkers to predict clinical outcome of immunotherapy.^5^
- Systematic evaluation of immune regulation and modulation.^6^
- Novel technologies and emerging biomarkers for personalized cancer immunotherapy.^7^
- Society for Immunotherapy of Cancer clinical and biomarkers data sharing resource document: Volume I—conceptual challenges.^8^
- Society for Immunotherapy of Cancer clinical and biomarkers data sharing resource document: Volume II—practical challenges.^9^

#### Technology primers

- Immune monitoring technology primer: immunosequencing.^10^
- Immune monitoring technology primer: the enzyme-linked immunospot (Elispot) and fluorospot assay.^11^
- Immune monitoring technology primer: single cell network profiling (SCNP).^12^
- Immune monitoring technology primer: flow and mass cytometry.^13^
- Immune monitoring technology primer: clinical validation for predictive markers.^14^
- Quantitative real-time PCR assisted cell counting (qPACC) for epigenetic-based immune cell quantification in blood and tissue.^15^
- nCounter PanCancer Immune Profiling Panel.^16^
- Immune monitoring technology primer: protein microarray (‘seromics’).^17^
- Multiplexed tissue biomarker imaging.^18^
- Immune monitoring technology primer: immunoprofiling of antigen-stimulated blood.^19^
- Immune technology primer: whole exome sequencing for neoantigen discovery and precision oncology.^20^
- Biomarkers immune monitoring technology primer: Immunoscore Colon.^21^

The current source document, which was developed by the SITC Biomarkers Committee of 2018–2019 to support biomarker research for various immunotherapeutic strategies, is an update from the cancer immunotherapy biomarker resource document published by SITC in 2011.

- SITC/iSBTc cancer immunotherapy biomarkers resource document: online resources and useful tools - a compass in the land of biomarker discovery.^22^

Since then, there have been revolutionary advances in the cancer immunotherapy field, highlighted by the successful clinical development of immune checkpoint inhibitors (ICIs) and genetically engineered cellular therapies. The management of a growing list of cancers has been transformed by immunotherapy, exemplified by the 5-year survival of more than 50% of patients with stage IV melanoma treated by combination checkpoint inhibitors. Immunotherapy was named *Science* magazine’s breakthrough of the year in 2013, and Dr James Allison and Dr Tasuku Honjo received the 2018 Nobel Prize for their contributions to the development of checkpoint inhibitors to treat patients with cancer. Despite this excitement, challenges remain, with low response rates in the majority of tumor types and the unique profile of immune-related adverse events (irAEs), which are hard to manage. Due to this conundrum, the utilization of biomarkers to prognosticate about patients’ overall cancer outcomes (regardless of therapy) or to predict response and toxicity from the effect of a therapeutic intervention, especially immunotherapy, is warranted. Both prognostic biomarkers (such as expression levels of programmed death-ligand 1 (PD-L1) and PD-L2 to predict survival outcomes in patients) and predictive biomarkers of response and toxicity are dealt with due to this urgent need, and these biomarkers are key to successful immunotherapy development, which is in the midst of an explosion of innovation. As demarcated by the National Cancer Institute (NCI) Dictionary of Cancer Terms, a biomarker is defined as:

A biological molecule (molecular marker and signature molecule) found in blood, other body fluids, or tissues that is a sign of a normal or abnormal process, or of a condition or disease. A biomarker may be used to see how well the body responds to a treatment for a disease or condition.

Thus, this document provides comprehensive tools and resources with supporting publications to summarize current information, facilitate biomarker discovery and validation, and discuss assays in development for clinical use. It encompasses topics of assay standardization and harmonization, novel biomarker discovery, regulatory agency guidelines for diagnostics, and new and emerging technologies. An attempt has been made to include many pertinent products, resources, and publications. However, as the field of immune biomarkers is growing very rapidly, it is impossible to provide an exhaustive list of all relevant products, resources and publications. A summary table of online resources is provided in the last section.

### Recently approved cancer immunotherapies—a breakthrough

#### Immune checkpoint inhibitors

In 2011, the first ICI, ipilimumab, an immune cell cytotoxic T lymphocyte-associated protein-4 (CTLA-4)-targeting monoclonal antibody, was approved by the FDA to treat patients with advanced melanoma based on two pivotal phase III clinical trials. Ipilimumab functions in the priming phase of T cell activation by inhibiting the immune suppressive CTLA-4 checkpoint and allows antitumor T cells to be activated and released from lymphoid tissue.

- Improved survival with ipilimumab in patients with metastatic melanoma.^23^
- Ipilimumab plus dacarbazine for previously untreated metastatic melanoma.^24^

Subsequently, in 2014, programmed cell death-1 (PD-1)-targeting monoclonal antibodies were approved, beginning with pembrolizumab for advanced or unresectable melanoma based on the KEYNOTE-001, KEYNOTE-002, and KEYNOTE-006 trials; and nivolumab based on the CheckMate 037, CheckMate 067, and CheckMate 069 trials. These antibodies strengthen antitumor immunity by releasing the ‘brakes’ that cause the immune suppression of effector T cells in the tumor microenvironment (TME). These antibodies have been subsequently approved in the treatment of more than 10 malignancies, spanning from Hodgkin lymphoma to head and neck carcinoma, with inspiring durability of response, resulting in widespread clinical application. A newer anti-PD-1 antibody, cemiplimab (2018), was approved for cutaneous squamous cell carcinoma based on Study 1540.

- Anti-programmed-death-receptor-1 treatment with pembrolizumab in ipilimumab-refractory advanced melanoma: a randomised dose-comparison cohort of a phase 1 trial.^25^
- Pembrolizumab vs ipilimumab in advanced melanoma.^26^
- Pembrolizumab vs investigator-choice chemotherapy for ipilimumab refractory melanoma (KEYNOTE-002): a randomised, controlled, phase 2 trial.^27^
- Nivolumab versus chemotherapy in patients with advanced melanoma who progressed after anti-CTLA-4 treatment (CheckMate 037): a randomised, controlled, open-label, phase 3 trial.^28^
- Nivolumab and ipilimumab versus ipilimumab in untreated melanoma.^29^
- Combined nivolumab and ipilimumab or monotherapy in untreated melanoma.^30^
- Development of PD-1 and PD-L1 inhibitors as a form of cancer immunotherapy: a comprehensive review of registration trials and future considerations.^31^
- PD-1 blockade with cemiplimab in advanced cutaneous squamous cell carcinoma.^32^

In addition, PD-L1 ICIs such as atezolizumab (2016), avelumab (2017), and durvalumab (2017) were approved by the FDA based on clinical trials POPLAR and OAK (both for non-small cell lung cancer, NSCLC), Study 1108 (refractory urothelial carcinoma), and JAVELIN (metastatic Merkel cell carcinoma). These antibodies target PD-L1 expressed by tumor cells to thwart the immunosuppression exerted by the interactions of PD-1 on T cells with PD-L1 on tumor cells.

- Atezolizumab versus docetaxel for patients with previously treated non-small-cell lung cancer (POPLAR): a multicentre, open-label, phase 2 randomised controlled trial.^33^
- Atezolizumab versus docetaxel in patients with previously treated nonsmall-cell lung cancer (OAK): a phase 3, open-label, multicentre randomized controlled trial.^34^
- IMFINZI prescribing information.^35^
- Durvalumab after chemoradiotherapy in stage III non-small-cell lung cancer.^36^
- Avelumab, an anti-programmed death-ligand 1 antibody, in patients with refractory metastatic urothelial carcinoma: results from a multicenter, phase Ib study.^37^

These ICIs are associated with durable responses, a hallmark of immunotherapy, and further increased overall survival (OS) and progression-free survival (PFS) compared with chemotherapies and targeted therapies in a subset of patients. On the other hand, many patients/tumor types do not respond to these interventions. This leads to combinatorial approaches among these ICIs and with other established agents such as chemotherapy, radiation, and molecularly targeted therapeutics. With the identification of more immune checkpoint molecules (eg, lymphocyte-activation gene 3 (LAG3), T cell immunoglobulin and mucin domain 3 (TIM3), B7-H3 (also known as CD276), V-domain Ig suppressor of T cell activation (VISTA) and adenosine A2A receptor (A2AR)), new options for single and combined therapy regimens with already approved ICIs are being explored in many ongoing clinical trials. However, irAEs can be severe, with unpredictable patterns of occurrence. For the continued successful development of cancer immunotherapies, biomarkers predicting response, resistance mechanisms, immune-related toxicities, and hyperprogression are paramount.

- Combining immune checkpoint inhibitors: established and emerging targets and strategies to improve outcomes in melanoma.^38^

#### Adoptive cell therapy

In 2017, the first anti-CD19 chimeric antigen receptor (CAR) T cell product, tisagenlecleucel (Kymriah), was approved for the treatment of pediatric and young adult patients with relapsed and/or refractory B cell precursor acute lymphoblastic leukemia, based on the ELIANA trial. This was followed by the second anti-CD19 CAR, axicabtagene ciloleucel (Yescarta), which was approved based on the ZUMA-1 trial for the treatment of adult patients with relapsed or refractory large B cell lymphoma. Despite great promise, treatment-related toxicities, relapse due to loss of CD19 on tumor cells, and lack of specificity (eg, targeting CD19 that is also expressed by other cells such as follicular dendritic cells) remain important issues. Therefore, identifying patients who would benefit from the therapy and preventing or managing unwanted toxicity, along with additional development of CAR T cell therapeutics for successful clinical application, are warranted.

- Tisagenlecleucel in children and young adults with B-cell lymphoblastic leukemia.^39^
- Axicabtagene ciloleucel CAR T-cell therapy in refractory large B-cell lymphoma.^40^

#### Cancer vaccines

Provenge (sipuleucel-T) is a cancer vaccine consisting of autologous dendritic cells loaded with a prostate tumor antigen, prostatic acid phosphatase (PAP), along with other peripheral blood mononuclear cells (PBMCs). It was the first therapeutic cancer vaccine approved by the FDA in 2010, based on three double-blind, randomized phase III studies on asymptomatic/minimally symptomatic metastatic castration-resistant prostate cancer and finally on the IMPACT trial reported by Kantoff PW *et al*.^41^ SITC produced a clinical practice guideline on this treatment approach for prostate cancer. Again, the real challenges with Provenge, apart from production issues, are the single-antigen targeting approach and methodological variation in detecting PAP as a biomarker to determine treatment options.

- Placebo-controlled phase III trial of immunologic therapy with sipuleucel-T (APC8015) in patients with metastatic, asymptomatic hormone refractory prostate cancer.^42^
- Integrated data from 2 randomized, double-blind, placebo-controlled, phase 3 trials of active cellular immunotherapy with sipuleucel-T in advanced prostate cancer.^43^
- Sipuleucel-T immunotherapy for castration-resistant prostate cancer.^41^
- The Society for Immunotherapy of Cancer consensus statement on immunotherapy for the treatment of prostate carcinoma.^44^

#### Oncolytic viral immune therapy

Oncolytic viruses (OVs) embody a new class of therapeutic agents that facilitate antitumor responses by combining selective tumor cell killing and the induction of systemic antitumor immunity. There are three OVs that have received regulatory approval: Rigvir in Latvia, Georgia, and Armenia; Oncorine H101 in China; and talimogene laherparepvec (T-VEC, Imlygic) in the USA. These consist of an echovirus, an adenovirus, and a herpes simplex-1 virus, respectively. Owing to the approval of T-VEC for treatment of melanoma in 2015 by the FDA, based on data from a pivotal phase III trial (OPTiM) in patients with advanced melanoma, oncolytic viral therapy has been accepted as a standard immunotherapy in the USA. However, the benefit of single-agent T-VEC delivered intralesionally is marginal in patients with visceral metastases. In order to enhance response rates, newer generations of OVs such as HF10 (canerpaturev, C-REV) and CVA21 (CAVATAK) are being tested as monotherapies and in combination with ICIs. Important hurdles for OV therapy are the presence of pre-existing antibodies to these viruses in vaccinated or seropositive patients, and the intact innate responses of tumor cells or immune cells in the TME that affect viral replication.

- OPTIM trial: a phase III trial of an oncolytic herpes virus encoding GM-CSF for unresectable stage III or IV melanoma.^45^
- Talimogene laherparepvec improves durable response rate in patients with advanced melanoma.^46^
- The emerging role of oncolytic virus therapy against cancer.^47^

A summary of the immunotherapies discussed above may be found in table 1.

**Table 1:** Recently approved immunotherapies

Drug name	Type of agent	Target	Supporting study	Year approved	FDA-approved use on first approval
Ipilimumab	ICI	CTLA-4	NCT00094653	2011	Melanoma
Pembrolizumab	ICI	PD-1	KEYNOTE-001 (NCT01295827)	2014	Melanoma
Nivolumab	ICI	PD-1	CheckMate 037 (NCT01721746)	2014	Melanoma
Cemiplimab	ICI	PD-1	Study 1423 (NCT02383212), Study 1540 (NCT02760498)	2018	Cutaneous squamous cell carcinoma
Atezolizumab	ICI	PD-L1	NCT02108652	2016	Urothelial carcinoma
Avelumab	ICI	PD-L1	JAVELIN Merkel 200 (NCT02155647)	2017	Merkel cell carcinoma
Durvalumab	ICI	PD-L1	Study 1108 (NCT01693562)	2017	Urothelial carcinoma
Tisagenlecleucel	CAR T cell	CD19	ELIANA (NCT02435849)	2017	B cell precursor acute lymphoblastic leukemia
Axicabtagene ciloleucel	CAR T cell	CD19	ZUMA-1 (NCT02348216)	2017	Large B cell lymphoma
Sipuleucel-T	Vaccine	Prostatic acid phosphatase	IMPACT (NCT00065442)	2010	Castration-resistant prostate cancer
Talimogene laherparepvec	Oncolytic virus	Tumor cells	OPTiM (NCT00769704)	2015	Melanoma

CAR T cell, chimeric antigen receptor T cell; CTLA-4, cytotoxic T lymphocyte-associated protein-4; FDA, Food and Drug Administration; ICI, immune checkpoint inhibitor; PD-1, programmed cell death protein-1; PD-L1, programmed death-ligand 1.

### Biomarkers of immune response and clinical outcome in patients with cancer

As highlighted above, immunotherapy has radically transformed the standard of cancer treatment but suffers from low frequency of benefit due to the complexity of resistance mechanisms. In order to increase the clinical efficacy of immunotherapy, combinations of immunotherapeutic agents and standard therapies are being developed. In this regard, the characterization and monitoring of immune responsiveness during immunotherapy treatment are important to understand the mechanisms of action of these therapeutic regimens, to optimize patient stratification and selection for combination strategies, and to monitor and predict treatment-related toxicities. Recent reviews by Pilla L and Maccali C,^48^ Darvin P *et al*,^49^ Lu S *et al*,^50^ and Wang Y *et al*^51^ focus on correlation of clinical responsiveness with different immunomonitoring strategies such as circulating immune cells (including absolute leukocyte count (Weide B *et al*,^52^ Martens A *et al*,^53^ Subrahmanyam PB *et al*^54^)), TME-associated immune cells, soluble serum markers, host microbiome, PD-L1 overexpression, neoantigens, and genetic and epigenetic signatures. A more detailed discussion on different biomarkers is provided in the last part of this section.

- Immune profiling of cancer patients treated with immunotherapy: advances and challenges.^48^
- Immune checkpoint inhibitors: recent progress and potential biomarkers.^49^
- Comparison of biomarker modalities for predicting response to PD-1/PD-L1 checkpoint blockade: a systematic review and meta-analysis.^50^
- Modulation of gut microbiota: a novel paradigm of enhancing the efficacy of programmed death-1 and programmed death ligand-1 blockade therapy.^51^
- Myeloid-derived suppressor cells predict survival of patients with advanced melanoma: comparison with regulatory T cells and NY-ESO-1- or melan-A-specific T cells.^52^
- Baseline peripheral blood biomarkers associated with clinical outcome of advanced melanoma patients treated with ipilimumab.^53^
- Distinct predictive biomarker candidates for response to anti-CTLA-4 and anti-PD-1 immunotherapy in melanoma patients^54^

### Biomarkers of immune-related adverse events and correlation with clinical response

As highlighted above, several factors have been shown to have potential as biomarkers for tumor response to ICIs, but factors which can predict irAEs are less common. IrAEs are diverse and vary according to the ICI agent. In this regard, a recent review by Nakamura,^55^ which highlights recent advances in the understanding of biomarkers for tumor response and the occurrence of irAEs in patients with cancer treated with ICIs, is valuable. Although controversial, the correlation of severe irAEs with clinical efficacy has been reported in NSCLC, head and neck, and other cancers. In a 2019 meeting abstract presentation, a higher rate of objective response and lower progression and death rates were reported in a cohort of patients with urothelial cancer who experienced irAEs. In another meeting report, the development of irAEs was associated with clinical benefit for patients with advanced gastric cancer receiving nivolumab monotherapy. Further studies with larger numbers of patients and longer follow-up are needed to validate these findings, in addition to developing biomarker-based assays to predict the development of irAEs.

- Do immune-related adverse events correlate with response to immune checkpoint inhibitors?.^56^
- Association between immune-related adverse events (irAEs) and clinical outcomes (CO) in advanced urothelial cancer patients (pts) treated with immunotherapy (IO).^57^
- Correlation between immune-related adverse events and prognosis in patients with gastric cancer treated with nivolumab.^58^
- Biomarkers for immune checkpoint inhibitor-mediated tumor response and adverse events.^55^

### Quantifying the antitumor immune response

Tumor development, or response to immunotherapy, dynamically shapes the composition and function of the immune response. The following select recent reviews and original research papers explore some of the current immunotherapies being assessed in patients with cancer and describe the experimental tools available for monitoring their antitumor immune response prior to or during treatment.

- Quantifying the anti-tumor immune response in patients receiving immunotherapy.^59^
- Comprehensive intrametastatic immune quantification and major impact of immunoscore on survival^60^
- Association between expression level of PD1 by tumor-infiltrating CD8^+^ T cells and features of hepatocellular carcinoma.^61^
- The development, function, and plasticity of the immune macroenvironment in cancer.^62^
- Enhanced adaptive immune responses in lung adenocarcinoma through natural killer cell stimulation^63^
- Quantifying antigen-specific T cell responses when using antigen-agnostic immunotherapies.^64^

### The development and validation of immunotherapy biomarkers

Recent comprehensive reviews by the SITC Biomarkers Committee (Masucci GV *et al*^3^ and Dobbin KK *et al*^4^) deal with considerations for preanalytical, analytical, and clinical validation of biomarkers to predict responses to cancer immunotherapy. Gnjatic S *et al*^5^ describe baseline immune-related biomarkers and how those biomarkers could predict the clinical outcome of immunotherapy. Lastly, the challenges to developing valuable immuno-oncology biomarkers are outlined by Mehnert JM *et al*.^65^

- Validation of biomarkers to predict response to immunotherapy in cancer: volume I—pre-analytical and analytical validation.^3^
- Validation of biomarkers to predict response to immunotherapy in cancer: volume II—clinical validation and regulatory considerations.^4^
- Identifying baseline immune-related biomarkers to predict clinical outcome of immunotherapy.^5^
- The challenge for development of valuable immuno-oncology biomarkers.^65^

### Data harmonization efforts for biomarker discovery

#### CIMAC/CIDC network

In 2017, the US NCI funded the CIMAC and the CIDC, as part of the Cancer Moonshot program. The CIMAC constitutes four academic centers (Dana-Farber Cancer Institute, MD Anderson Cancer Center, Mount Sinai School of Medicine, and Stanford University Medical School), which are responsible for providing standardized, analytically validated, state-of-the-art immune monitoring assays for early-stage NCI trials involving cancer immunotherapy. The CIDC, housed at the Dana-Farber Cancer Institute, will create a database for aggregation and integrated analysis of CIMAC data, biomarker discovery, and sharing with the scientific community. For more information, see:

- https://cimac-network.org/66

In partnership with the CIMAC/CIDC network, a consortium of 11 biopharmaceutical companies was created by the Foundation for the National Institutes of Health. Called PACT, this public–private collaboration will extend the CIMAC/CIDC activities to include additional non-NCI clinical trials, with the goal of accelerating biomarker discovery in immuno-oncology. More information can be found at:

- https://fnih.org/what-we-do/programs/partnership-for-accelerating-cancer-therapies^67^

#### Assay standardization and harmonization

Standardization and harmonization are two integral parts for controlling the performance of biomarker assays to allow for consistency and comparability of results. Standardization of an operational assay procedure addresses each single variable of an assay, and is hence restricted to a specific application and laboratory or laboratory network that follows the established standard operating procedure (SOP) for the defined application. Standardization is a prerequisite for assay validation. Reviews and guidelines on biomarker assay standardization are numerous. More information is provided within the specific assay subsections of this document.

Assay harmonization addresses the major limitations of assay standardization, which are twofold: (1) explorative biomarker assays often require procedural adaptations, and (2) countless minute assay variables exist, which are impossible to standardize across a large number of laboratories. Harmonization focuses on key protocol variables that influence the assay outcome, and their alignment across laboratories and SOPs. Assay harmonization efforts are currently underway in the CIMAC network for cytometry by time of flight (CyTOF), single parameter and multiplex immunohistochemistry (IHC), RNA-seq, and whole exome sequencing (WES), as these assays are performed across multiple CIMAC centers. An outline for achieving biomarker assay harmonization has been given.

- Harmonization of immune biomarker assays for clinical studies.^68^

### Sample collection: technical considerations for processing, storage, and shipment of tumor samples for immunological studies

Regardless of the assay type (eg, detection of DNA, RNA, or protein expression), quality of data is largely influenced by the quality of the biospecimens used (eg, blood including isolated serum/plasma and mononuclear immune cells, body fluid, tissue such as tumors, and so on). Factors influencing the quality of biospecimens are often referred to as preanalytical variables, which involve sample processing, storage, and shipment. A comprehensive list of guidelines on the best practices for biorepositories and biospecimens is available from the NCI, which includes comprehensive checklists created by the College of American Pathologists (CAP). The International Society for Biological and Environmental Repositories (ISBER) also publishes Best Practices for Repositories, which reflects the collective experience of repository professionals.

- https://biospecimens.cancer.gov/bestpractices/69
- https://www.cap.org/laboratory-improvement/accreditation/accreditation-checklists^70^
- https://www.isber.org/page/BPR^71^

The following reviews discuss the most up-to-date understanding of preanalytical variables:

- Preanalytical challenges - time for solutions.^72^
- The root causes of pharmacodynamic assay failure^73^
- Tumor pre-analytics in molecular pathology: impact on protein expression and analysis.^74^
- Understanding preanalytical variables and their effects on clinical biomarkers of oncology and immunotherapy.^75^

### Reporting of biomarker data in clinical trials and publications

To allow an objective evaluation of biomarker data, the reporting has to follow standards of conformity and transparency to support the rigor required for reproducibility and confidence in the data. The REMARK (REporting recommendations for tumor MARKer prognostic studies) guidelines for prognostic tumor marker studies have been widely accepted.^76^ Reporting recommendations for specific biomarker assays exist as minimal information guidelines, and are further addressed in this document, for example, for microarray assays,^77^ T cell assays,^78^ and flow cytometry assays.^79^ A review commentary about achieving greater reproducibility and credibility of early clinical biomarker studies, including data reporting, has recently been published.^80^

- REporting recommendations for tumor MARKer prognostic studies (REMARK).^76^
- Minimum information about a microarray experiment (MIAME)—toward standards for microarray data.^77^
- T cell assays and MIATA: the essential minimum for maximum impact.^78^
- MIFlowCyt: the minimum information about a flow cytometry experiment.^79^
- In pursuit of greater reproducibility and credibility of early clinical biomarker research.^80^

#### Conclusions

For new biomarker discovery, using validated assays is important, especially in larger clinical trials. Harmonization of assays across sites is difficult but needed whenever multiple sites are expected to generate comparable data. Control of preanalytical variables is key to the success of biomarker assays, and standardized reporting is required for effective evaluation and data reuse.

### Novel biomarker discovery: immunotherapy biomarker useful literature review

#### PD-L1 expression

Anti-PD-1 checkpoint inhibitors have revolutionized cancer care. Six PD-(L)1-specific antibodies including nivolumab (Opdivo; anti-PD-1), pembrolizumab (Keytruda; anti-PD-1), atezolizumab (Tecentriq; anti-PD-L1), durvalumab (Imfinzi; anti-PD-L1), avelumab (Bavencio; anti-PD-L1), and cemiplimab (Libtayo; anti-PD-1) have been approved by the US FDA in specific tumor indications; clinical benefit from this class of agents, however, is restricted to a subset of patients. Assessment of tumor PD-L1 expression by IHC was a rational choice for biomarker development, and a number of PD-L1 IHC assays, including 28-8 pharmDx (Agilent), 22C3 pharmDx (Agilent), Ventana SP142 (Roche Diagnostics), Ventana SP263 (Roche Diagnostics) and Dako/Agilent 73-10 (Agilent), have been developed to support patient selection and diagnostic strategies for nivolumab, pembrolizumab, atezolizumab, durvalumab, and avelumab, respectively. However, it has become apparent that PD-L1 protein is an imprecise biomarker in predicting clinical benefit from PD-(L)1-specific antibodies. While multiple studies have found a positive correlation between tumor PD-L1 expression and clinical efficacy of anti-PD-1 blockade, others have detected no association. Many patients with PD-L1-negative tumors also derive durable clinical benefit from anti-PD-1 inhibitors. The contradictory data from correlative studies around PD-L1 IHC are attributable to multiple factors, including the inducible nature and intertumor and intratumor heterogeneity of PD-L1 expression and technical variations, such as different detection antibodies and assay platforms used, archival versus fresh tumor tissue, type and duration of tissue fixation, non-standardized criteria, and various cut-off levels to define positive expression.

- Comparing and contrasting predictive biomarkers for immunotherapy and targeted therapy of NSCLC.^81^
- Monitoring immune-checkpoint blockade: response evaluation and biomarker development.^82^
- Mechanism-driven biomarkers to guide immune checkpoint blockade in cancer therapy.^83^
- What does PD-L1 positive or negative tumors mean?^84^
- Predictive biomarkers for checkpoint inhibitor-based immunotherapy.^85^
- PD-L1 expression as a predictive biomarker in cancer immunotherapy.^86^

Assessment of PD-L1 expression on tumor versus immune cells adds another level of complexity. In certain tumor types (eg, squamous cell carcinoma of the head and neck (SCCHN), melanoma, breast cancer and renal cell carcinoma (RCC)), PD-L1 is expressed on the surface of both tumor and immune cells (macrophages, dendritic cells, and activated T cells), whereas in others such as colorectal cancer (CRC) and gastric carcinoma, PD-L1 expression is predominantly seen on tumor-infiltrating immune cells. In certain tumor types, clinical activity of PD-(L)1 inhibitors is associated with PD-L1 on immune rather than tumor cells. Therefore, scoring algorithms based on PD-L1 expression in both the tumor and immune cell compartments have been established.

- Predictive correlates of response to the anti-PD-L1 antibody MPDL3280A in cancer patients.^87^
- Atezolizumab versus docetaxel for patients with previously treated non-small-cell lung cancer (POPLAR): a multicentre, open-label, phase 2 randomised controlled trial.^33^
- Atezolizumab in patients with locally advanced and metastatic urothelial carcinoma who have progressed following treatment with platinum-based chemotherapy: a single-arm, multicentre, phase 2 trial.^88^
- Clinical utility of the combined positive score for programmed death ligand-1 expression and the approval of pembrolizumab for treatment of gastric cancer.^89^
- Establishing a complementary diagnostic for anti-PD-1 immune checkpoint inhibitor therapy.^90^

In contrast to complementary tests (which assist in risk-benefit analysis but are not required for the use of a therapy), companion diagnostic (CDx) tests are required for use with a specific therapy to identify patients who are most likely to benefit from that therapy. For pembrolizumab, for example, the 22C3 pharmDx assay has been approved by the FDA as a CDx test to help identify eligible patients with NSCLC, gastric or gastroesophageal junction (GEJ), cervical, and urothelial carcinoma. PD-L1 expression in NSCLC is determined using the Tumor Proportion Score (TPS), which is the percentage of viable tumor cells showing partial or complete membrane PD-L1 staining at any intensity. An NSCLC specimen is considered PD-L1-positive for the purposes of first-line treatment with pembrolizumab monotherapy if the TPS is ≥ 1% of tumor cells. PD-L1 protein expression in gastric/GEJ, cervical, SCCHN, esophageal squamous cell carcinoma, and urothelial carcinomas is determined by the Combined Positive Score (CPS), which is defined as the percentage of PD-L1-positive tumor and immune cells relative to the total number of tumor cells. The sample is considered to have PD-L1 expression if it has a CPS ≥1 for gastric/GEJ, SCCHN, and cervical carcinomas and ≥10 for urothelial cell carcinoma (UCC) and esophageal squamous cell carcinoma. The FDA also approved the Ventana SP142 assay as a CDx test to select patients with (1) locally advanced or metastatic UCC who are cisplatin-ineligible for single-agent treatment with atezolizumab or unresectable/locally advanced and (2) metastatic triple-negative breast cancer (TNBC) for combination treatment with atezolizumab and nanoparticle albumin-bound paclitaxel. The SP142 assay determines tumor PD-L1 positivity as PD-L1 stained tumor-infiltrating immune cells of any intensity covering ≥1% or 5% of the tumor area in TNBC and UCC, respectively.

- https://www.fda.gov/drugs/resources-information-approved-drugs/fda-grants-accelerated-approval-pembrolizumab-advanced-gastric-cancer^91^
- https://www.fda.gov/drugs/resources-information-approved-drugs/fda-updates-prescribing-information-keytruda-and-tecentriq^92^
- https://www.fda.gov/drugs/resources-information-approved-drugs/fda-approves-pembrolizumab-advanced-cervical-cancer-disease-progression-during-or-after-chemotherapy^93^
- https://www.fda.gov/Drugs/InformationOnDrugs/ApprovedDrugs/ucm633065.htm^94^
- https://www.fda.gov/drugs/fda-expands-pembrolizumab-indication-first-line-treatment-nsclc-tps-1^95^
- https://www.fda.gov/drugs/resources-information-approved-drugs/fda-approves-pembrolizumab-first-line-treatment-head-and-neck-squamous-cell-carcinoma^96^
- https://www.fda.gov/drugs/resources-information-approved-drugs/fda-approves-pembrolizumab-advanced-esophageal-squamous-cell-cancer^97^

Currently, there is no standardized approach for PD-L1 testing. Significant heterogeneity was reported for the available PD-L1 IHC tests, with different cut-off points and testing standards, which makes interpretation of the PD-L1 expression data across various clinical trials very challenging. In the Blueprint PD-L1 IHC Assay Comparison Project (an industrial–academic collaborative partnership), as well as in a similar National Comprehensive Cancer Network (NCCN) project, different PD-L1 IHC assays, including 28-8 pharmDx, 22C3 pharmDx, Ventana SP142, Ventana SP263, and Dako/Agilent 73-10, were evaluated to provide information on their analytical and clinical comparability. The results of this effort demonstrated comparable analytical results for the 22C3, 28-8, and SP263 assays, but differences were noted with regard to the SP142 and 73-10 assays for determining TPS on tumor cells. A greater variability between tests was observed when PD-L1 expression was analyzed on immune cells. Although the data suggest possible interchangeability of some PD-L1 IHC tests (but not for assessment of PD-L1 expression on immune cells), some discordance in the results was apparent, and the interchangeable use of these assays may result in misclassification of PD-L1 status for some patients. A recent meta-analysis suggests that the FDA-approved PD-L1 IHC assays that were designed and approved for a different purpose may not be interchangeable with each other. In contrast, well-designed, fit-for-purpose PD-L1 laboratory-developed IHC tests appear to achieve higher accuracy than the FDA-approved PD-L1 IHC assays when both are compared with an appropriate designated reference standard.

- PD-L1 immunohistochemistry assays for lung cancer: results from phase I of the Blueprint PD-L1 IHC assay comparison project.^98^
- Programmed death ligand-1 immunohistochemistry testing: a review of analytical assays and clinical implementation in non-small cell lung cancer.^99^
- PD-L1 immunohistochemistry comparability study in real-life clinical samples: results of Blueprint phase 2 project.^100^
- A prospective, multi-institutional, pathologist-based assessment of 4 immunohistochemistry assays for PD-L1 expression in non-small cell lung cancer. 101
- Automated image analysis of NSCLC biopsies to predict response to anti-PD-L1 therapy.^102^
- “Interchangeability” of PD-L1 immunohistochemistry assays: a meta-analysis of diagnostic accuracy.^103^

#### Prognostic prediction of PD-L1 and PD-L2 expression

A meta-analysis study suggests that PD-L1 overexpression is related to poor OS in patients with cervical cancer and poor PFS in Asian patients with cervical cancer. This study also suggests that PD-L1 expression is a promising prognostic indicator for cervical cancer. In this scenario, PD-L1 assay validation is critical for its utility in routine clinical practice.

- Elevated PD-L1 expression predicts poor survival outcomes in patients with cervical cancer.^104^

Although PD-L2 is more confined to antigen-presenting cells, its expression has been discovered in many tumor types owing to induction by stimuli in the TME. A recent meta-analysis revealed that high PD-L2 expression in solid tumors, especially in hepatocellular carcinoma (HCC), predicts tumor metastasis and unfavorable prognosis after surgery. In this scenario, it is unknown what additional correlation might be achieved by combining PD-L2 with PD-L1 measurement.

- Correlation between PD-L2 expression and clinical outcome in solid cancer patients: a meta-analysis.^105^

##### Conclusions

PD-L1 IHC has demonstrated clinical utility by allowing patient selection and enrichment for clinical benefit from single-agent treatment with anti-PD-1 checkpoint inhibitors. A number of PD-L1 IHC tests were independently codeveloped to support specific anti-PD-(L)1 programs, and the lack of standardization between these IHC requires harmonization of these assays in the clinic, as well as consensus on the scoring algorithms and cut-off levels to define positive PD-L1 status across various tumor types. While PD-L1 IHC tests allow for enrichment of patients who are likely to derive clinical benefit from anti-PD-(L)1 agents, their clinical utility is less clear in the context of combination immunotherapies (eg, nivolumab/ipilimumab, angio-immunotherapy, and chemoimmunotherapy) which, based on currently available data, appear to be efficacious irrespective of tumor PD-L1 status.

#### Tumor mutational burden

Human tumors harbor a varying number of somatic mutations collectively referred to as tumor mutational burden (TMB). TMB has become a useful biomarker in immuno-oncology following the demonstration that a correlation between high TMB and clinical efficacy of ICIs exists across multiple tumor types. Initial interest in TMB was triggered by two exploratory studies of WES data obtained from patients with melanoma; a correlation between TMB and the magnitude of clinical benefit in ipilimumab (anti-CTLA-4)-treated patients was observed.

- Genetic basis for clinical response to CTLA-4 blockade in melanoma.^106^
- Genomic correlates of response to CTLA-4 blockade in metastatic melanoma.^107^

In addition, a high response rate to anti-PD-1 checkpoint inhibitors was observed in desmoplastic melanoma, a subtype of melanoma that has very high median mutational burden.

- High response to PD-1 blockade in desmoplastic melanoma.^108^

Similar observations were made in patients with NSCLC treated with anti-PD-1 antibodies.

- Mutational landscape determines sensitivity to PD-1 blockade in non-small cell lung cancer.^109^

The clinical relevance of TMB was further demonstrated in a study of mismatch repair-deficient tumors (frequently detected as tumors with high microsatellite instability (MSI-H)); these tumors exhibited a markedly increased mutational load and displayed high objective response rates after anti-PD-1 blockade.

- PD-1 blockade in tumors with mismatch repair deficiency.^110^

Based on the results of larger randomized and non-randomized clinical trials (Checkmate-012, Checkmate-569, Checkmate-227), TMB has emerged as a potential biomarker predictive of clinical benefit in patients with NSCLC treated with combined ICIs (in this case, nivolumab/ipilimumab). Ten mutations per megabase was identified as an optimal cut-off level to define the NSCLC patient population with high TMB. However, while high TMB appears to be associated with improved PFS, a correlation between TMB and OS in patients treated with combined immune checkpoint blockade has not been demonstrated.

- Genomic features of response to combination immunotherapy in patients with advanced non-small cell lung cancer.^111^
- Nivolumab plus ipilimumab in lung cancer with a high tumor mutational burden.^112^
- First-line nivolumab plus ipilimumab in advanced non-small-cell lung cancer (CheckMate 568): outcomes by programmed death ligand 1 and tumor mutational burden as biomarkers.^113^
- Nivolumab plus ipilimumab in advanced non-small-cell lung cancer.^114^

An association of TMB with response to checkpoint blockade was also demonstrated in patients with small cell lung cancer treated with nivolumab and ipilimumab.

- Tumor mutational burden and efficacy of nivolumab monotherapy and in combination with ipilimumab in small-cell lung cancer.^115^

Furthermore, investigators analyzed genomic data of >1600 patients with advanced cancer treated with some type of ICI, whose tumors were subjected to the targeted next-generation sequencing (NGS) test Memorial Sloan Kettering-Integrated Mutation Profiling of Actionable Cancer Targets (MSK-IMPACT) established at Memorial Sloan Kettering Cancer Center. For most, but not all, tumor types, higher somatic TMB (highest 20% in each histology) correlated with improved survival in patients receiving ICIs across multiple cancer types; however, based on these data one universal definition of high TMB appears to be unlikely.

- Tumor mutational load predicts survival after immunotherapy across multiple cancer types.^116^

In line with these data, another study of whole exomes of microsatellite stable tumors (n=294) concluded that TMB has insufficient predictive power to differentiate tumor responses from progressive disease, and therefore additional molecular correlates should be taken into consideration.

- Genomic correlates of response to immune checkpoint blockade in microsatellite-stable solid tumors.^117^

Based on the results of the KEYNOTE clinical trials spanning 22 tumor types and >300 patients treated with pembrolizumab, investigators from Merck & Co also concluded that the TMB and T cell-inflamed gene expression profiles (GEPs) exhibited only modest correlation and were independently predictive of clinical outcome (see ‘Immune gene expression signatures’ section for an examination of T cell-inflamed GEPs). However, when analyzed jointly, TMB and GEP were capable of defining a patient population (TMB-high/GEP-high) deriving maximum clinical benefit from pembrolizumab.

- Pan-tumor genomic biomarkers for PD-1 checkpoint blockade-based immunotherapy.^118^

Not all somatic mutations are alike in their potential to generate neoantigens. Frameshift insertion and deletion (indel) mutations are believed to be a rich source of immunogenic neoantigens. Indel burden may help explain some discrepancy in the data for TMB and ICI response in specific tumor indications, including RCC, which has a good rate of response to ICIs (~25%), although most patients with RCC have low TMB. RCC had the highest frequency of indel mutations among 19 cancer types analyzed, and frameshift indel mutations were found to be ~3 times more immunogenic than non-synonymous mutations; the relationship between indel burden and clinical efficacy of ICIs needs to be investigated further.

- Insertion-and-deletion-derived tumour-specific neoantigens and the immunogenic phenotype: a pan-cancer analysis.^119^
- Tumor exome analysis reveals neoantigen-specific T-cell reactivity in an ipilimumab-responsive melanoma.^120^
- Checkpoint blockade cancer immunotherapy targets tumour-specific mutant antigens.^121^
- Mutational landscape determines sensitivity to PD-1 blockade in non-small cell lung cancer.^109^

Somatic copy number alterations, such as amplifications and deletions, represent another complexity of tumor-specific genomic aberrations that may affect the tumor immune microenvironment and clinical efficacy of ICIs.

- Molecular and genetic properties of tumors associated with local immune cytolytic activity.^122^
- Integrated molecular analysis of tumor biopsies on sequential CTLA-4 and PD-1 blockade reveals markers of response and resistance.^123^

WES of matched tumor and normal tissue samples is a gold standard of TMB analysis. However, it requires high coverage sequencing of ~50 Mb of genomic content and is technically and is operationally challenging for routine use in clinical practice. Targeted NGS panels that use hybridization-capture methodologies such as the FoundationOne CDx (F1CDx by Foundation Medicine) and MSK-IMPACT assays that target 324 and 468 cancer-related genes, respectively, have been used to assess TMB in tumor biopsy samples; compared with WES, they have a shorter turnaround time and are more cost-effective for clinical sample analysis.

- https://www.foundationmedicine.com/genomic-testing/foundation-one-cdx^124^
- https://www.mskcc.org/msk-impact^125^
- Comprehensive cancer-gene panels can be used to estimate mutational load and predict clinical benefit to PD-1 blockade in clinical practice.^126^
- Tumor mutational load predicts survival after immunotherapy across multiple cancer types.^116^

TMB quantifies the mutations found in a tumor. Currently, there are no standards for calculating and reporting TMB. Similar to efforts with harmonizing PD-L1 assays by IHC, the harmonized measurement of TMB is ongoing, with the goal of helping reduce potential variability and optimizing its use. The TMB Harmonization Working Group has issued its plan for upcoming analyses of human tumor cells. The working group will create a universal reference standard using WES and identify sources of potential variability. To date, the working group has reviewed publicly available data from The Cancer Genome Atlas (TCGA) and identified sources of variability between TMB calculated using WES and various targeted panels used in the clinic. The work is ongoing, and phase I results will be reported at an upcoming meeting.

- Development of tumor mutation burden as an immunotherapy biomarker: utility for the oncology clinic.^127^

#### Friends of Cancer Research TMB Harmonization Working Group

- https://www.focr.org/TMB^128^
- https://www.focr.org/news/friends-cancer-research-announces-launch-phase-ii-tmb-harmonization-project^129^

With the advent of methods enabling analysis of tumor-derived DNA in the circulation (ctDNA), an approach commonly referred to as liquid biopsy, it may be possible to assess TMB by ctDNA sequencing. Analysis of ctDNA using the Guardant Health NGS panel targeting 54–70 genes revealed that the total number of mutations detected in ctDNA positively correlated with clinical benefit from ICIs in a clinical trial of 69 patients representing 23 different cancer types. An obvious question is whether TMB in ctDNA could accurately reflect TMB evaluated in tumor biopsy samples. A blood-based platform using the aforementioned FoundationOne CDx assay was capable of measuring TMB in plasma samples (blood TMB, or bTMB) in two large randomized clinical trials (POPLAR and OAK); bTMB correlated with TMB measured in tumor biopsy samples in NSCLC, and therefore has the potential to identify patients who derive clinical benefit from anti-PD-L1 treatment (such as atezolizumab). Furthermore, preliminary results from the MYSTIC phase III trial of first-line durvalumab with or without tremelimumab (anti-CTLA-4) versus platinum-based chemotherapy in NSCLC indicate that in patients with high bTMB (≥20 mut/Mb), identified by the GuardantOMNI platform, treatment with durvalumab and tremelimumab was associated with both OS and PFS benefit.

- Hypermutated circulating tumor DNA: correlation with response to checkpoint inhibitor-based immunotherapy.^130^
- Blood-based tumor mutational burden as a predictor of clinical benefit in non-small-cell lung cancer patients treated with atezolizumab.^131^
- Clinical potential of circulating tumour DNA in patients receiving anticancer immunotherapy.^132^
- Tumor mutational burden (TMB) as a biomarker of survival in metastatic non-small cell lung cancer (mNSCLC): blood and tissue TMB analysis from MYSTIC, a phase III study of first-line durvalumab ± tremelimumab vs chemotherapy.^133^

While emerging data for TMB as a biomarker predictive of efficacy of ICIs look encouraging, it is apparent that TMB assessment needs to be standardized across platforms and laboratories. Several key factors should be taken into consideration to enable the comparison of TMB data across various platforms: depth and length of sequencing reads; choice of aligners, variant callers, and filters used; and preanalytical variability due to inconsistency in sample collection and processing, input material quality and quantity, fixation methodology, and library preparation should also be addressed.

- Tumor mutational burden standardization initiatives: recommendations for consistent tumor mutational burden assessment in clinical samples to guide immunotherapy treatment decisions.^134^
- Development of tumor mutation burden as an immunotherapy biomarker: utility for the oncology clinic.^127^

#### Defective mismatch repair

In an interesting study with a single tumor type, the presence of a defective mismatch repair system and the presence of tumor-infiltrating lymphocytes (TILs) could be linked to better outcomes from novel immune-based therapies in patients with advanced gastric cancer.

- Mismatch repair deficiency may affect clinical outcome through immune response activation in metastatic gastric cancer patients receiving first-line chemotherapy.^135^
- PD-1 blockade in tumors with mismatch-repair deficiency.^110^

Using a database of more than 10000 tumors, immunogenomic analysis of data compiled by TCGA could serve as a resource to identify patients likely to respond to particular immunotherapies.

- The immune landscape of cancer.^136^

##### Conclusions

TMB and other genetic determinants have demonstrated the potential to make immune checkpoint therapy more precise. Clinical data in support of the predictive value of TMB in the context of ICIs are encouraging but not fully conclusive, and challenges remain. It remains to be seen if tumor and/or bTMB can help identify patients who are likely to benefit from combination immunotherapies, including, but not limited to, angio-immunotherapy and chemoimmunotherapy combinations. Additionally, the variability in the current methods of TMB assessment may complicate therapeutic decisions in the clinic. This highlights the need for standardization and harmonization of TMB analysis and reporting across assays and laboratories.

#### Tumor-infiltrating T cells

T cells are the most important effector cells in the antitumor immune response. There is compelling evidence on the prognostic significance of intratumoral CD8^+^ T cell density across multiple tumor types. The location, density, and phenotype of tumor-infiltrating immune cells are three important parameters of the intratumoral immune contexture. The concept of Immunoscore was developed by quantifying and qualifying the T cell infiltrate in the tumor core as well as at the invasive tumor margins to predict tumor recurrence and survival in patients with stage I–III colon cancer, using a four-point scale. The potential utility of this scoring approach in other tumor types (eg, melanoma, NSCLC) is being evaluated:

- The immune contexture in human tumours: impact on clinical outcome.^137^
- International validation of the consensus Immunoscore for the classification of colon cancer: a prognostic and accuracy study.^138^
- Immunoscore and immunoprofiling in cancer: an update from the melanoma and immunotherapy bridge.^139^
- Assessing PDL-1 and PD-1 in non-small cell lung cancer: a novel Immunoscore approach.^140^

Since the antitumor activity of anti-CTLA-4 and anti-PD-1 inhibitors is attributed at least in part to the reinvigoration of dysfunctional T cells in the TME, both the density and location of intratumoral T cells have also emerged as potential predictive biomarkers for ICIs. While baseline density does not appear to correlate with clinical activity of ipilimumab, pre-existing CD8^+^ (but not CD4^+^) T cell infiltration at the invasive tumor margin and within the tumor core is associated with response to anti-PD-1 therapy in patients with melanoma. In both anti-CTLA-4-treated and anti-PD-1-treated patients with melanoma, increases in intratumoral T cells while on treatment were associated with clinical activity, while a higher proximity of CD68^+^ myeloid cells to CD8^+^ T cells was documented in non-responders to anti-PD-1. Three main phenotypes were described in the context of anti-PD-1 pathway blockade: (1) the immune-desert phenotype (absence of immune cells within or around the tumor), (2) the immune-excluded phenotype (immune cells surrounding but not penetrating the tumor), and (3) the inflamed phenotype (immune cells penetrating the tumor, but presumably non-functional).

- A prospective phase II trial exploring the association between tumor microenvironment biomarkers and clinical activity of ipilimumab in advanced melanoma.^141^
- PD-1 blockade induces responses by inhibiting adaptive immune resistance.^142^
- Analysis of immune signatures in longitudinal tumor samples yields insight into biomarkers of response and mechanisms of resistance to immune checkpoint blockade.^143^
- Predictive correlates of response to the anti-PD-L1 antibody MPDL3280A in cancer patients.^87^
- Elements of cancer immunity and the cancer-immune set point.^144^

In-depth immunophenotypic analyses of TILs or the TME have shown correlation of the following T cell phenotypes with clinical benefit from checkpoint inhibitors or cellular therapy in patients with melanoma or NSCLC: (1) baseline frequency of tumor-infiltrating CD8^+^ T cells coexpressing PD-1 and CTLA-4 (PD-1^hi^ CTLA-4^hi^ cells) and exhibiting an exhausted phenotype; (2) high absolute levels of PD-1 on CD8^+^ TILs; (3) a ‘dormant’ TIL phenotype (CD3^hi^ GzmB^lo^ Ki67^lo^); (4) increased cytolytic activity (cytolytic score defined as the geometric mean of Perforin 1 and Granzyme A mRNA expression); (5) reduction of non-conventional CD4^+^ Foxp3^−^ PD-1^+^ T cells (4PD-1^hi^ cells) on anti-PD-1 treatment; (6) ratio of memory-like TCF7^+^ (also known as TCF1) to CD39^+^ TIM3^+^ cells within CD8^+^ T cells; (7) high frequency of TCF1^+^ PD-1^+^ CD8^+^ T cells; (8) improved metabolic fitness and low mitochondrial membrane potential of TCF1^+^ stem cell memory T cells (Tscm cells); and (9) high frequency of tissue-resident memory T cells (Trm cells) that express the integrin CD103. The CD8^+^ T cells expanded in treated tumors displayed an exhausted, terminally differentiated phenotype, while the corresponding CD4^+^ T cell population displayed a T helper 1 (Th1)-like effector phenotype. There is an increased frequency of Th1-like T cells in melanoma samples treated by anti-CTLA-4 compared with those treated by anti-PD-1 antibodies.

- Tumor immune profiling predicts response to anti-PD-1 therapy in human melanoma.^145^
- A transcriptionally and functionally distinct PD-1^+^ CD8^+^ T cell pool with predictive potential in non-small-cell lung cancer treated with PD-1 blockade.^146^
- A dormant TIL phenotype defines non-small cell lung carcinomas sensitive to immune checkpoint blockers.^147^
- Tumor and microenvironment evolution during immunotherapy with nivolumab.^148^
- Non-conventional inhibitory CD4^+^Foxp3^-^PD-1^hi^ T cells as a biomarker of immune checkpoint blockade activity.^149^
- Defining T cell states associated with response to checkpoint immunotherapy in melanoma.^150^
- Checkpoint blockade immunotherapy induces dynamic changes in PD-1^-^CD8^+^ tumor-infiltrating T cells.^151^
- Subsets of exhausted CD8^+^ T cells differentially mediate tumor control and respond to checkpoint blockade.^152^
- Intratumoral Tcf1^+^PD-1^+^CD8^+^ T cells with stem-like properties promote tumor control in response to vaccination and checkpoint blockade immunotherapy.^153^
- Mitochondrial membrane potential identifies cells with enhanced stemness for cellular therapy.^154^
- Single-cell profiling of breast cancer T cells reveals a tissue-resident memory subset associated with improved prognosis.^155^
- Tissue-resident memory features are linked to the magnitude of cytotoxic T cell responses in human lung cancer.^156^
- Tissue-resident memory T cells at the center of immunity to solid tumors.^157^
- Resident memory T cells, critical components in tumor immunology.^158^
- Distinct cellular mechanisms underlie anti-CTLA-4 and anti-PD-1 checkpoint blockade.^159^

Emerging data from a recent work that used T cell receptor (TCR) sequencing coupled with functional studies of tumor-infiltrating T cells suggest that many tumor-infiltrating T cells are not reactive against tumor cells, and are in fact specific for epitopes related to viruses (Epstein-Barr virus (EBV), cytomegalovirus (CMV), or influenza virus) rather than tumor antigens. These bystander CD8^+^ T cells may exhibit phenotypes that overlap with tumor-specific cells, but lack CD39 expression. Furthermore, tumor-infiltrating CD103^+^ CD39^+^ CD8^+^ T cells that display an exhausted Trm phenotype showed an enrichment for tumor-specific cells with a distinct tumor-specific TCR repertoire; contrary to what their exhausted phenotype might suggest, they efficiently kill autologous tumor cells in a major histocompatibility complex (MHC) class I-dependent manner, and their frequencies positively correlate with OS in patients with SCCHN.

- Low and variable tumor reactivity of the intratumoral TCR repertoire in human cancers.^160^
- Bystander CD8^+^ T cells are abundant and phenotypically distinct in human tumour infiltrates.^161^
- Co-expression of CD39 and CD103 identifies tumor reactive CD8 T cells in human solid tumors.^162^

In two types of patients with cancer, melanoma and NSCLC, those whose tumors exhibited increased inflammatory gene transcripts with high circulating CD4^+^ and CD8^+^ central memory T cell (Tcm) to effector T cell ratios had longer PFS.

- Circulating T cell subpopulations correlate with immune responses at the tumor site and clinical response to PD1 inhibition in non-small cell lung cancer.^163^

##### Conclusions

Assessments of T cell density, location, and phenotype in baseline and on-treatment tumor samples provide important insights into the role of these cells in patients with cancer and immune checkpoint therapy. It is apparent that complex immune monitoring approaches and robust computational solutions are needed to better characterize the tumor immune contexture.

#### Immune gene expression signatures

High-throughput gene expression profiling has enabled the development of transcriptomic profiles in predicting response or resistance to ICIs. Numerous gene expression signatures have been evaluated for specific tumor types or across multiple indications; however, their clinical utility needs to be further explored.

A pan-cancer 18-gene T cell-inflamed signature associated with clinical benefit of pembrolizumab was developed by Merck using GEPs of baseline tumor samples spanning nine tumor types and 220 patients. This gene signature is predominantly represented by interferon (IFN)-γ-responsive genes related to antigen presentation, chemokine expression, cytolytic activity, and adaptive immune resistance, and has been deployed in ongoing clinical trials of pembrolizumab. An eight-gene T-effector/IFN-γ (Teff/IFN-γ) gene expression signature defined by CD8A, GZMA, GZMB, IFN-γ, EOMES, CXCL9, CXCL10, and TBX21 was developed by Genentech. This signature was indicative of pre-existing tumor immunity and was associated with clinical benefit from atezolizumab in a second-line treatment of NSCLC. In line with these data, investigators from MedImmune (now AstraZeneca) identified a four-gene IFN-γ^+^ signature comprising IFN-γ, CD274, LAG3, and CXCL9, which was associated with clinical efficacy of durvalumab in NSCLC and UCC.

- IFN-γ-related mRNA profile predicts clinical response to PD-1 blockade.^164^
- Atezolizumab versus docetaxel for patients with previously treated non-small-cell lung cancer (POPLAR): a multicentre, open-label, phase 2 randomised controlled trial.^33^
- Interferon gamma messenger RNA signature in tumor biopsies predicts outcomes in patients with non-small cell lung carcinoma or urothelial cancer treated with durvalumab.^165^

A transcriptional signature related to innate anti-PD-1 resistance (IPRES) was identified in patients with melanoma. The IPRES signature is driven by increased expression of genes involved in the regulation of mesenchymal transition, cell adhesion, extracellular matrix remodeling, angiogenesis, and wound healing, and these transcriptomic changes are also seen in patients with melanoma after treatment with mitogen-activated protein kinase (MAPK) pathway inhibitors, suggesting overlapping mechanisms of resistance to MAPK and anti-PD-1 inhibitors. High scores of a pan-fibroblast transforming growth factor-β (TGF-β) response signature were associated with lack of clinical benefit from atezolizumab in UCC with a T cell-excluded phenotype. Another epithelial-mesenchymal transition (EMT)-related gene expression signature helped define outcomes of patients with UCC with high intratumoral T cell density treated by nivolumab. In addition, the clinical efficacy of atezolizumab in metastatic RCC is inversely correlated with a high myeloid inflammation signature defined by upregulation of interleukin (IL)-6, CXCL1, CXCL2, CXCL3, CXCL8, and PTGS2 identified within Teff^hi^ tumors, while a combination of atezolizumab and bevacizumab (anti-vascular endothelial growth factor) appeared to be efficacious in this patient population (Teff^hi^ Myeloid^hi^).

- Genomic and transcriptomic features of response to anti-PD-1 therapy in metastatic melanoma.^166^
- TGFβ attenuates tumour response to PD-L1 blockade by contributing to exclusion of T cells.^167^
- EMT- and stroma-related gene expression and resistance to PD-1 blockade in urothelial cancer.^168^
- Clinical activity and molecular correlates of response to atezolizumab alone or in combination with bevacizumab versus sunitinib in renal cell carcinoma.^169^

Immunophenoscore was developed using data from TCGA for 20 tumor types based on the expression of genes related to MHC molecules, costimulatory/coinhibitory molecules, effector T cells, and immunosuppressive cell subsets, and was associated with survival in 12 tumor types and predicted response to checkpoint inhibitors in two independent cohorts.

- Pan-cancer immunogenomic analyses reveal genotype-immunophenotype relationships and predictors of response to checkpoint blockade.^170^

A computational tumor immune dysfunction and exclusion (TIDE) framework was developed using publicly available data from >33,000 human tumor samples with transcriptome and patient survival information by testing the effects of interactions among the candidate genes with either cytotoxic T cells or immunosuppressive cell signatures on the risk of death. TIDE provides signatures of both T cell dysfunction in immunologically hot tumors and T cell exclusion in cold tumors. When applied to pretreatment transcriptomic data from patients with melanoma subsequently treated with ICIs, TIDE outperformed other predictive biomarkers tested, including PD-L1 expression, TMB, and IFN-γ signature.

- Signatures of T cell dysfunction and exclusion predict cancer immunotherapy response.^171^

Another predictive score, IMPRES (immunopredictive score), was developed from gene expression changes of 15 rational pairwise relationships between immunoinhibitory and immunostimulatory genes associated with spontaneous immune-mediated regression of neuroblastoma and extrapolated to other tumor types (eg, melanoma). High IMPRES scores were found to define immunologically hot tumors and predict clinical outcomes in patients with melanoma treated with different ICIs.

- Robust prediction of response to immune checkpoint blockade therapy in metastatic melanoma.^172^

Single-cell RNA sequencing (scRNA-seq) profiling identified a transcriptional resistance program in malignant cells that is associated with T cell exclusion and immune evasion. This cyclin-dependent kinase 4/6 (CDK4/6)-dependent signature was detected prior to immunotherapy and predicted clinical responses to anti-PD-1 therapy in an independent cohort of 112 patients with melanoma.

- A cancer cell program promotes T cell exclusion and resistance to checkpoint blockade.^173^

Endogenous retroviral elements (ERVs), integrated into human DNA over the past 100 million years of primate evolution, constitute ~8.5% of the human genome and are normally transcriptionally silent; transcription of ERV sequences can result in the activation of RNA sensing pathways and subsequent production of type I and/or II interferons (IFNs). There is also evidence of tumor-specific human ERV (hERV) epitopes that can be translated and presented on MHC class I molecules to the cognate tumor-reactive T cell clones. A transcriptomic hERV signature has shown prognostic value in patients with RCC.

- Molecular and genetic properties of tumors associated with local immune cytolytic activity.^122^
- Endogenous retroviral signatures predict immunotherapy response in clear cell renal cell carcinoma.^174^
- ERVmap analysis reveals genome-wide transcription of human endogenous retroviruses.^175^

Furthermore, gene expression profiling of both tumor samples and peripheral T cells enabled identification of shared and non-overlapping transcriptomic changes in patients treated with anti-PD-1 and anti-CTLA-4 inhibitors.

- Analysis of immune signatures in longitudinal tumor samples yields insight into biomarkers of response and mechanisms of resistance to immune checkpoint blockade.^143^
- Combination therapy with anti-CTLA-4 and anti-PD-1 leads to distinct immunologic changes in vivo.^176^

#### Useful reviews

- Mechanism-driven biomarkers to guide immune checkpoint blockade in cancer therapy.^83^
- Implementing TMB measurement in clinical practice: considerations on assay requirements.^177^

#### Guidelines and meeting reports

- Method validation and measurement of biomarkers in nonclinical and clinical samples in drug development: a conference report.^178^

### Regulatory agency guidelines for diagnostics

The FDA has approved two types of CDx tests for some immuno-oncology therapeutics and indications: PD-L1 IHC assays and MSI analyses. There are FDA-approved tests reporting metrics of TMB and *B2M/JAK/LKB1* mutations. Regulatory agency approval and guidance on the use of these tests may differ. Key agencies to monitor include FDA (USA), European Medicines Agency (EMA; European Union), Pharmaceuticals and Medical Devices Agency (PMDA; Japan), and National Medical Products Administration (NMPA; China). Importantly, the guidelines may change and should be monitored for the latest updates.

The FDA issued a draft guidance document to address the potential challenges when multiple CDx tests are in use for the same disease indication. For instance, an additional biopsy and/or a different CDx needs to be obtained to have additional treatment options, which is not optimal. With the draft guidance (references below), manufacturers may expand current CDx tests by submitting a premarket approval, supplement, or a new ‘510(k) application, as appropriate, to expand the labeling to broaden the indication for use with a specific group or class of oncology products in the same disease’.

Agencies post their guidance documents, roadmaps, and/or approved medical devices on their websites.

#### USA: FDA

Example list of cleared or approved CDx devices from the FDA:

- https://www.fda.gov/medicaldevices/productsandmedicalprocedures/invitrodiagnostics/ucm301431.htm^179^

FDA guidance issued April 2020:

- https://www.fda.gov/regulatory-information/search-fda-guidance-documents/developing-and-labeling-vitro-companion-diagnostic^180^
- https://www.fda.gov/NewsEvents/Newsroom/PressAnnouncements/ucm627745.htm^181^

#### Europe: EMA

EMA presentations on new guidance, October 2018:

- https://www.ema.europa.eu/documents/presentation/presentation-interface-between-medicinal-product-medical-devices-development-update-ema_en.pdf^182^

EMA Competent Authorities for Medical Devices Implementation Taskforce Roadmap 2017:

- https://www.camd-europe.eu/wp-content/uploads/2018/05/NEWS_171107_MDR-IVDR_RoadMap_v1.3-1.pdf^183^

EMA concept paper on evolving landscape for biomarkers and CDx (August 2017):

- https://www.ema.europa.eu/documents/scientific-guideline/concept-paper-predictive-biomarker-based-assay-development-context-drug-development-lifecycle_en.pdf^184^

#### Japan: PMDA

Website of approvals:

- https://www.pmda.go.jp/english/review-services/reviews/approved-information/drugs/0002.html^185^

#### Other consortia, collaboration projects, and meeting groups

- Immunoscore task force.^186^
- PACT: a public–private partnership to aid standardization of immune therapy biomarkers.
- Parker Institute for Cancer Immunotherapy’s ‘TESLA’ (Tumor NeoantigEN SeLection Alliance) collaborative project: neoantigen selection and the TESLA consortium.^187^
- CIDC and CIMAC/CIDC network.
- American Association for Cancer Research Project GENIE (Genomics Evidence Neoplasia Information Exchange).^188^

#### Conclusions

The gene expression data sets generated in clinical trials of ICIs provide important insights into the mechanisms underlying the antitumor effects of this class of agents, and allow for both qualitative and quantitative assessment of the tumor immune microenvironment at baseline and on treatment with immunomodulatory agents. Transcriptomic profiling represents a powerful and promising approach to predict sensitivity and resistance to ICIs and identify new targets in immuno-oncology. While numerous lines of evidence demonstrate the potential of gene expression signatures to enrich for patients who are likely to benefit from single-agent treatment with ICIs, transcriptomic profiling may also help identify patient populations for combination immunotherapies, as exemplified by the aforementioned data for the myeloid gene expression signature and clinical activity of atezolizumab + bevacizumab versus atezolizumab in RCC. Additional transcriptomic data are needed to help differentiate patients with cancer who would be appropriate candidates for anti-PD-(L)1 monotherapy and for combination immunotherapies.

## New and emerging technologies for biomarker discovery

Biomarker discovery for immunotherapy is challenging, as the efficacy of the treatment relies not only on the characteristics of the tumor cells, but also the host’s immune system, as well as the interaction of the immune system and the tumor cells in the dynamic TME. In addition, each patient may have a unique combination of features that determine their sensitivity to a particular treatment. Therefore, biomarker research for immunotherapy needs to go beyond the tumor itself and explore the TME and the host. In this section, we introduce different technology platforms that can be useful in biomarker discovery. For tumor immunogenicity, intrinsic resistance and neoantigen-focused research and nucleic acid-based platforms, including genomic, transcriptomic, epigenetic, and PCR/hybridization techniques, are instrumental. For dynamic changes in the tumor immune contexture and the host’s immune susceptibility, proteomic platforms ranging from ELISA to mass cytometry, along with multiplex imaging technologies, can be helpful. Overall, the development of reliable biomarkers that can predict the efficacy of different immunotherapeutic agents and their combination is key to the success of extending the benefit of immunotherapy to a majority of patients with cancer.

### Genomic biomarker discovery

#### Whole exome sequencing

The protein-coding sequences of a gene are called exons, and all the combined exons in a genome are referred to as the exome. With existing technology, 95% of the human exome can be sequenced. Therefore, the term ‘exome sequencing’ is more accurate than the term ‘whole-exome sequencing (WES)’. It is noteworthy that while the human exome comprises all coding nuclear DNA sequences, mitochondrial DNA is not included. The exome represents less than 2% of the human genome, but contains about 85% of known disease-related variants, establishing exome sequencing as a cost-effective alternative to whole genome sequencing (WGS). Exome sequencing using exome enrichment can efficiently identify coding variants across a wide range of applications, including population genetics, genetic disease, and cancer studies. However, exome sequencing techniques have non-standardized, highly variable coverage, including regions of the exome that are refractory to being accurately sequenced, such as genes containing a pseudogene, highly repetitive coding regions, large deletions, and duplications. Therefore, it is likely that some clinically significant mutations would be missed by exome sequencing due to inefficient capture of relevant exons:

- Targeted capture and massively parallel sequencing of 12 human exomes.^189^
- Educational materials—genetic testing: current approaches.^190^

Cancer is driven by genomic events, and different sets of genetic aberrations can characterize individual cancers. The use of high-throughput sequencing, including exome sequencing, to identify those changes can guide the identification of effective therapies currently available or still in clinical trials.

- Personalized oncology through integrative high-throughput sequencing: a pilot study.^191^
- Whole-exome sequencing of metastatic cancer and biomarkers of treatment response.^192^

Clinical implementation of genomic data to inform therapy necessitates that clinicians interpret the patient’s genomic profile, including both tumor and germline DNA. Currently, only a limited number of genomic markers in specific cancer settings have shown strong evidence of differential response to specific therapies, as these are targetable mutations with approved or investigational therapies. For many other known genomic alterations, there are no data or insufficient data to support routine clinical implementation of biomarker-based therapy.

- A decision support framework for genomically informed investigational cancer therapy.^193^
- Precision oncology in the age of integrative genomics.^194^

##### Conclusions

The rapid advances in availability and affordability of NGS technology provide the potential to include WES in routine, genomically informed, personalized cancer therapy. WES is a rational option because most known driver mutations occur in exons, and thus WES is thought to be an efficient method to identify a broad array of possible targetable mutations. However, WES could miss mutations outside the exons that lead to aberrant gene activity and protein production. Development of broadly accessible, comprehensive, and regularly updated databases that link observed genomic changes to clinically actionable phenotypes, and continued education of clinicians and patients about advantages and limitations, will greatly facilitate broader clinical implementation of this approach.

#### TCR sequencing and clonality

Human T cells mature in the thymus from hematopoietic progenitors, gain the ability to recognize foreign antigens, and provide protection against a vast array of pathogens.

A complex molecular mechanism in T cells based on somatic recombination leads to the expression of highly polymorphic surface receptors, the TCRs, and provides the immune system with functional plasticity. TCR sequencing (TCR-seq) produces large numbers of short DNA sequences covering key regions of the TCR coding sequence, allowing quantification of T cell diversity at high resolution. Reduced cost of high-throughput sequencing technologies has enabled the identification of immune response signatures based on sequence analysis. In consequence, high-throughput TCR-seq has been established as a tool to analyze antigen specificity, clonality, and diversity of T lymphocytes:

- Linking T-cell receptor sequence to functional phenotype at the single-cell level.^195^
- Overview of methodologies for T-cell receptor repertoire analysis.^196^

The characterization of the TCR repertoire through TCR-seq is of great scientific and potential clinical relevance because it accurately describes T cell dynamics in a wide range of diseases, including infection, autoimmune diseases, and malignancies:

- Tumor-infiltrating lymphocytes in colorectal tumors display a diversity of T cell receptor sequences that differ from the T cells in adjacent mucosal tissue.^197^
- TCR sequencing facilitates diagnosis and identifies mature T cells as the cell of origin in CTCL.^198^
- Characteristics of tumor infiltrating lymphocyte and circulating lymphocyte repertoires in pancreatic cancer by the sequencing of T cell receptors.^199^
- A new high-throughput sequencing method for determining diversity and similarity of T cell receptor (TCR) α and β repertoires and identifying potential new invariant TCR α chains.^200^

High-throughput TCR-seq is rapidly evolving, and numerous validated procedures for clonotype identification and TCR repertoire analysis exist. However, no gold standard method has been established in the field. A number of platforms are available, including DNA-based (eg, Adaptive), RNA-based (eg, iRepertoire), bulk TCR-seq, and single-cell TCR-seq (10X) technologies. Different approaches may be more applicable than others for different scientific purposes, but can be subject to possible method-specific biases. There are innovative approaches, such as combining TCR-seq with an assay for transposase-accessible chromatin analysis at the single cell level for information on TCR specificity and the epigenomic state of individual T cells, which will likely expand the academic and clinical utility of this technology:

- Single cell T cell receptor sequencing: techniques and future challenges.^201^
- Transcript-indexed ATAC-seq for precision immune profiling.^202^
- Quantifiable predictive features define epitope-specific T cell receptor repertoires.^203^
- Identifying specificity groups in the T cell receptor repertoire.^204^
- Using T cell receptor repertoires to understand the principles of adaptive immune recognition.^205^

##### Conclusions

TCR-seq, clonality, and repertoire analysis are valuable tools to help elucidate T cell biology in healthy individuals and pathological conditions, including cancer. It is being used not only to investigate mechanisms of immune-mediated diseases, but also to monitor immune responses to therapies, including immunotherapy. Sequencing the TCRs of thousands of cells in parallel is a powerful technology to dissect the complexity and diversity of the T cell response repertoire. Advances in single-cell technologies and corresponding data management can deliver accurate sequence information on paired alpha and beta chains of individual cells, and enable high-throughput TCR-seq as a routine tool for immune monitoring and biomarker development.

#### Epigenetic immune cell quantification with qPCR-based assisted cell counting (qPACC)

DNA-based, immune cell subset-specific epigenetic markers have recently been identified and can be used to differentiate leukocytes, lymphocytes, and other cell types of interest. These markers can also be used for epigenetic cell counting.

- DNA demethylation in the human FOXP3 locus discriminates regulatory T cells from activated FOXP3^+^ conventional T cells.^206^
- Quantitative DNA methylation analysis of FOXP3 as a new method for counting regulatory T cells in peripheral blood and solid tissue.^207^
- Epigenetic quantification of tumor-infiltrating T-lymphocytes.^208^

Relative cell numbers can be quantified using quantitative real-time PCR (qPCR) based on knowledge of unmethylated DNA regions of previously characterized cell types, in combination with bisulfite conversion (BSC). During BSC, unmethylated cytosines in DNA convert to uracil, but methylated cytosines are protected and remain unchanged. The resulting sequence changes are the foundation for developing differentiating primer and probe sets for qPCR on clinical samples. Different cell types relevant for immune monitoring during immunotherapy have been described, for example, Treg, CD3^+^, CD4^+^, and CD8^+^ T cells, B cells, natural killer (NK) cells, and neutrophils. In addition, several approaches to control, calibration, and quantification are used that allow the calculation of immune cell concentrations with qPCR-based assisted cell counting (qPACC), in a process referred to as epigenetic cell counting.

- Quantitative real-time PCR assisted cell counting (qPACC) for epigenetic-based immune cell quantification in blood and tissue.^15^
- Epigenetic immune cell counting in human blood samples for immunodiagnostics.^209^

The general stability of DNA as well as its methylation, in addition to the small sample volume needed, provide epigenetic-based assays the advantage of being less susceptible to challenges related to sample amount and quality, and permit the measurement of different immune cell subset frequencies without the need to count intact cells.

The immune cell subset-specific epigenetic markers are developed based on highly purified, fluorescence-activated cell sorting (FACS)-sorted cells, obtained from whole blood of healthy donors. Cancer is a disease affecting the DNA of patients, including, but not limited to, DNA strand breaks, gene duplication, and aberrant DNA methylation. In the immuno-oncology setting, this represents a theoretical obstacle to the successful use of this technology, since the specificity of the established cell type markers from healthy individuals could be compromised in cancer, precluding their use, at least in tumor-affected tissues.

- Demethylation of the *FOXP3* gene in human melanoma cells precludes the use of this epigenetic mark for quantification of Tregs in unseparated melanoma samples.^210^

##### Conclusions

There is a lack of peer-reviewed and published clinical studies using this technology during immunotherapy trials that independently demonstrate the consistency and validity of enumerating various subsets of lymphocytes in the peripheral blood of patients with cancer in comparison with fully validated, gold standard technologies like flow cytometry. However, it is theoretically an attractive methodology and has the potential to change research and clinical trial monitoring strategies. The cell type specificity of epigenetic-based qPACC assays is likely not a significant obstacle toward enumerating circulating lymphocytes in most immune oncology trial settings. The potential exception could be hematological malignancies, which would warrant particular attention to signals indicating issues with assay specificity. The use of epigenetics-based qPACC assays for the enumeration of different cell types in tumor tissue carries a theoretical and published risk of lacking specificity. In addition, it has no ability to provide relevant data on spatial immune cell distribution within a tumor, and therefore allows no direct comparison or correlation to classic or multiplex IHC.

### Microbiome sequencing

The human body is inhabited by countless microorganisms that live within diverse communities specific to each body site, including the skin, nose and mouth, eyes, and gastrointestinal and urogenital tracts. The human microbiome (or human microbiota) is referred to as the collection of micro-organisms which live on and in us, and comprised not just bacteria, but also fungi, protozoa, and viruses. The important role the microbiome plays in human health and disease, including oncology, is a broadly accepted fact today:

- The human microbiome: at the interface of health and disease.^211^

Impressive progress in high-throughput sequencing methods used by human genome research has benefitted the investigation of the microbiome greatly by enabling high-throughput microbial characterization in a culture-independent manner. The two most common methods of sequencing used to study the microbiome are 16 S rRNA sequencing and shotgun metagenomics.

- Genomic approaches to studying the human microbiota.^212^

The 16S rRNA is part of the 30 S subunit of prokaryotic ribosomes. The 16S ribosomal gene is understood to be present in all bacteria and contains regions that are highly variable between species that can be used to differentiate between different bacteria without having to sequence their entire genome. This approach permits targeting of only very specific regions of the genome, dramatically reducing the amount of sample and sequencing needed. The main disadvantage of this technology is that it can only identify and differentiate bacteria; it cannot be used to detect or differentiate viruses, fungi, or protozoa. There are different protocols and platforms available for 16S rRNA sequencing, and a more indepth analysis of platforms can be found in the following:

- A comprehensive benchmarking study of protocols and sequencing platforms for 16 S rRNA community profiling.^213^
- The madness of microbiome: attempting to find consensus “best practice” for 16 S microbiome studies.^214^

Shotgun metagenomic sequencing is the other approach most often used. During shotgun sequencing, all DNA within a complex sample are fragmented into very small pieces and then amplified and analyzed with NGS technology. It permits the study of the entire genomes of all the organisms present in a sample, including viruses, fungi, and protozoa. Shotgun metagenomics can give indications as to dominant gene pathways and functions, and are less susceptible to the biases that are inherent in targeted gene amplification.

- Shotgun metagenomics, from sampling to analysis.^215^

Different microorganisms and the microbiota, in general, are able to increase or alleviate carcinogenesis, alter sensitivity to cancer therapeutics, and influence response to immunotherapy. There are several areas of positive or negative contribution to carcinogenesis by microbes. They include changing the balance of host cell proliferation and death, altering immune system function, and influencing a host’s metabolism. Microbiota can act as an adjuvant, enhancing efficacy or attenuating toxicity of chemotherapies.

- Cancer and the microbiota.^216^
- The role of microbiota in cancer therapy.^217^
- Microbiome and anticancer immunosurveillance.^218^

A direct detrimental immunological effect has been shown, for example, in the inhibition of NK cell killing of various tumors by *Fusobacterium nucleatum*.

- Binding of the Fap2 protein of *Fusobacterium nucleatum* to human inhibitory receptor TIGIT protects tumors from immune cell attack.^219^

Efforts to target the microbiota in oncology settings should take into account that, during fecal microbiota transplantation (FMT), adverse events (including patient death) have been observed in attempted treatments of recurrent or refractory *Clostridium difficile* infections and other intestinal or extraintestinal disorders.

- Systematic review: adverse events of fecal microbiota transplantation.^220^

#### Conclusions

Studies have demonstrated the influence of the microbiome on carcinogenesis and response to therapy. However, it will require extensive research and resources to obtain reliable and clinically actionable information. The microbiota can vary considerably over time, between individuals, and in different areas of the body. Establishing clear distinctions in regard to the cause and effect of tumor-associated microbiota and concurrent changes in the microbiota is essential. The prognostic potential and theoretical value of therapeutic intervention toward the microbiota offer the exciting possibility of new tools to fight cancer, while making current therapies more effective and reducing side effects.

### Mitochondrial genome arrays

Mitochondrial DNA (mtDNA) genes encode proteins that work in conjunction with nuclear genes to form the respiratory chain complexes that represent the main energy production structures in cells. Because of its high susceptibility to mutations based on limited repair mechanisms existing (as compared with nuclear DNA), mtDNA has long been suspected to contribute to carcinogenesis. Since mtDNA lacks introns, mutations always affect coding sequences, and an accumulation of these mutations may lead to tumor formation. Research into the role of mtDNA mutations in cancer is advancing our understanding of their contribution to carcinogenesis and their potential value in cancer diagnosis and monitoring.

- Mitochondrial DNA mutations in human disease.^221^
- Human mitochondrial DNA: roles of inherited and somatic mutations.^222^
- Mitochondria and cancer.^223^
- How do changes in the mtDNA and mitochondrial dysfunction influence cancer and cancer therapy? Challenges, opportunities and models.^224^
- The landscape of mtDNA modifications in cancer: a tale of two cities.^225^

High-throughput mitochondrial sequencing arrays (eg, MitoChip by Affymetrix) are used in research and clinical studies for the identification of mtDNA markers associated with malignancies. Using the MitoChip technique, large numbers of mtDNA mutations have been found in human cancers, including CRC, head and neck cancer, bladder cancer, breast cancer, adenoid cystic carcinoma, sessile serrated adenoma, lung cancer, urinary bladder carcinomas, RCC, pancreatic cancers, ovarian carcinomas, gastric cancers, gliomas, and several other solid tumors.

- The human MitoChip: a high-throughput sequencing microarray for mitochondrial mutation detection.^226^
- MtDNA as a cancer marker: a finally closed chapter?^227^

The MD Anderson Cancer Center provides an online tool, called ‘The Cancer Mitochondrial Atlas (TCMA) data portal’, in order to assist mitochondria-related biological discoveries and clinical applications beyond mtDNA sequencing. This open-access data portal allows exploration of various types of molecular data:

- https://ibl.mdanderson.org/tcma/228

The TCMA consists of four modules: somatic mutations, nuclear transfer, copy number, and gene expression. International Cancer Genome Consortium (ICGC) WGS data are the basis for the first three modules and provide detailed annotations for the corresponding features of each cancer sample. TCGA RNA-seq data are the basis for the last module and provide an interactive interface through which operators can visualize the coexpression network. Operators can browse and query molecular data by cancer type and download the data for their own analysis.

#### Conclusions

The use of MitoChip to track mutations in mtDNA has been shown to be relevant in diverse cancer settings. However, its diagnostic, prognostic, and clinical value is still debated in the field based on several technology-related obstacles to standardization and relevant controls. New NGS approaches, combined with sophisticated data analysis to select mutations with likely functional relevance, could help to more systematically evaluate the potential role of mtDNA mutations in tumor biology in the future.

### Epigenomic biomarker discovery

DNA methylation, histone modifications, chromatin remodeling and spatial orientation, and post-transcriptional regulation influence gene expression and cellular phenotype without altering the nucleotide sequence of DNA. Collectively referred to as epigenomic signaling, these processes orchestrate cell development and differentiation, carcinogenesis, cancer progression, and resistance to therapy.

An overview of the history of epigenetics, various epigenetic processes, and their role in health and disease can be found in the following review.

- The molecular hallmarks of epigenetic control.^229^

For detailed reviews on epigenetic processes, the following collection is useful.

- https://www.cell.com/cell/collections/transcription-epigenetics^230^

Biomarkers that detect these epigenetic processes are crucial for diagnosis, prognostication, and therapeutic targeting. For example, the following review enumerates epigenomic biomarkers useful in the diagnosis and prognosis of hepatocellular carcinoma.

- Biomarkers: what role do they play (if any) for diagnosis, prognosis and tumor response prediction for hepatocellular carcinoma?^231^

In a study evaluating alternative promoter utilization in metastatic gastric cancer, higher levels of alternative promoter utilization predicted lower immunogenicity, cancer immunoediting, and evasion of immune checkpoint inhibition therapy, thus providing an epigenetic biomarker to predict response to immunotherapy.

- Epigenomic promoter alterations predict for benefit from immune checkpoint inhibition in metastatic gastric cancer.^232^

Epigenomic profiling has become automated, miniaturized, and reproducible, with the ability to resolve at the single cell level. Widely available epigenetic database and processing software have now made it possible to perform epigenomic profiling of tumors for biomarker discovery. This paper reviews the various methods of epigenomic biomarker discovery, their compatibility with sample preservation techniques, automation, reproducibility, and miniaturization.

- Genome-wide epigenomic profiling for biomarker discovery.^233^

This paper discusses the computational methods for assessing chromatin hierarchy.

- Computational methods for assessing chromatin hierarchy.^234^

#### ATAC-seq

Eukaryotic DNA is extensively packaged around histone proteins, forming nucleosomes, which are condensed into higher levels of packaging to allow chromatin to fit within the nucleus of a cell. Nevertheless, processes that allow ‘open’ chromatin states, which permit transcription factors and histone post-translational changes to influence gene expression, orchestrate active transcription.

Assay for transposase accessible chromatin with high-throughput sequencing, or ATAC-seq for short, is a method for mapping chromatin accessibility genome-wide. Hyperactive transposase Tn5 is used to cut and ligate adaptors for high-throughput sequencing of DNA in regions of high accessibility. This allows mapping of ‘open’ chromatin areas as well as nucleosome topology. The following paper describes ATAC-seq of lymphoblastoid cells.

- ATAC-seq: a method for assaying chromatin accessibility genome-wide.^235^

Significant chromatin heterogeneity can exist within a population of cells. Accuracy is enhanced when chromatin assays can be performed at a single cell level. The following paper talks about single-cell chromatin profiling.

- A rapid and robust method for single cell chromatin accessibility profiling.^236^

The profiling of chromatin of different cells within a cell population can inform cell subsets. The following papers describe single-cell ATAC-seq (scATAC-seq) in multiple cells to cluster them.

- Single-cell ATAC-seq: strength in numbers.^237^
- High-throughput chromatin accessibility profiling at single-cell resolution.^238^

Transcript-indexed ATAC-seq is a tool by which the TCR gene is sequenced along with ATAC-seq at the single cell level. Transcript-indexed ATAC-seq enables an analysis of the epigenetic landscape of a clonal T cell population and also enables discovery of regulatory pathways affecting T cell function.

- Transcript-indexed ATAC-seq for precision immune profiling.^202^

In this paper, the team has identified a programmable and a dysfunctional chromatin state in tumor-infiltrating T cells based on chromatin assays. They have identified the epigenetic processes associated with dysfunctional immune cells and surface biomarkers to identify reprogrammable T cells.

- Chromatin states define tumour-specific T cell dysfunction and reprogramming.^239^

Studies of chromatin states using ATAC-seq in immunology to reveal epigenetic heterogeneity, the mechanistic basis of T cell dysfunction, and distinct T cell subsets are as follows.

- Joint single-cell DNA accessibility and protein epitope profiling reveals environmental regulation of epigenomic heterogeneity.^240^
- Newly identified T cell subsets in mechanistic studies of food immunotherapy.^241^

Satpathy AT *et al*^242^ describe the use of scATAC-seq with a droplet-based method on a widely used single-cell 10X Chromium platform to discover cell types and regulatory DNA elements in complex tissues. They performed scATAC-seq using bone marrow and blood samples to characterize the chromatin landscape of cell subtypes and their differentiation trajectories. They then performed scATAC-seq on primary tumor tissue before and after treatment with PD-1 blockade. The authors demonstrate the ability to deconvolute the TME at the single cell level, revealing subpopulations of immune cells and malignant cells.

- Massively parallel single-cell chromatin landscapes of human immune cell development and intratumoral T cell exhaustion.^242^

The following paper describes the various methods used to assess chromatin accessibility, including the limitations, advantages, and specimen requirements of each technique: micrococcal nuclease sequencing (MNase-seq), DNase-seq, formaldehyde-assisted isolation of regulatory elements sequencing (FAIRE-seq), and ATAC-seq.

- Chromatin accessibility: a window into the genome.^243^

#### ChIP arrays (ChIP on chip) and ChIP-seq

The interaction of DNA with transcription factors and histones affects gene expression and cell phenotype. Chromatin immunoprecipitation (ChIP) is used widely to establish specific DNA–protein interactions. When ChIP is combined with whole genome DNA microarrays, the assay is known as a ChIP microarray or ChIP on chip. A genome-wide assessment of protein–DNA interactions was made possible through ChIP arrays and led to the discovery of epigenomic transcriptional regulation. Several tiling microarray platforms for common model organisms were commercially developed, and bioinformatics tools were generated to analyze data from those platforms. Some early descriptions of methods, applications, and analytical tools for ChIP on chip are referenced in the following.

- ChIP-chip: considerations for the design, analysis, and application of genome-wide chromatin immunoprecipitation experiments.^244^
- Chromatin immunoprecipitation for determining the association of proteins with specific genomic sequences in vivo.^245^
- Genome-wide profiling of PPARγ:RXR and RNA polymerase II occupancy reveals temporal activation of distinct metabolic pathways and changes in RXR dimer composition during adipogenesis.^246^
- rMAT-- an R/Bioconductor package for analyzing ChIP-chip experiments.^247^

More recently, massive parallel sequencing of DNA fragments crosslinked to protein has been made possible by a newly developed high-throughput sequencing technology. This technology, referred to as ChIP-seq, represents a large advancement in the study of DNA–protein interactions.

ChIP-seq has higher sensitivity and specificity than ChIP on chip and can be used to analyze any sequenced species, since it is not dependent on a microarray. It is also more cost-effective than ChIP on chip. The use of ChIP-seq in non-coding regions of the DNA has the potential to identify the biological role of single nucleotide polymorphisms (SNPs) associated with a disease state.

The following study compares ChIP on chip using the Agilent tiling microarray with ChIP-seq using the Illumina GAII.

- ChIP-chip vs ChIP-seq: lessons for experimental design and data analysis.^248^

The following describe ChIP-seq assays and bioinformatic tools for data analysis:

- Practical guidelines for the comprehensive analysis of ChIP-seq data.^249^
- ChIP-seq and beyond: new and improved methodologies to detect and characterize protein-DNA interactions.^250^
- Identifying and mitigating bias in next-generation sequencing methods for chromatin biology.^251^

#### Methylation arrays

In the human body, using the same genome, at least 200 distinct cell phenotypes can be created by means of epigenomic processes. An epigenetic imprint of cell types enhances cell recognition. In fact, the recent years have seen the identification of cell types based on epigenomic profiles by making use of reference methylation profiles of known cell types from the gene expression omnibus (GEO). In one such study, investigators performed epigenetic deconvolution of SCCHN samples from the TCGA, identifying different cell types in the tumors by histoepigenetic means. By identifying various cell populations in the tumors as immune, cancer, and epidermal cells, the investigators were able to epigenetically profile the distinct types of SCCHN by differential methylation analysis:

- Histoepigenetic analysis of HPV- and tobacco-associated head and neck cancer identifies both subtype-specific and common therapeutic targets despite divergent microenvironments.^252^

Several methylated genes serve as biomarkers of disease diagnosis, prognosis, and therapeutic targeting. With the availability of epigenome-wide methylation arrays, a much more amplified biomarker discovery platform is available.

The computational analysis of DNA methylation data involves multiple steps of data processing. The following papers describe the use of Illumina-based Infinium DNA methylation BeadChip assays, which are considered to be the gold standard method for DNA methylation analysis. These assays provide quantitative measurement of DNA methylation levels in a predetermined set of cytosine residues using a microarray format. With advancing capabilities and demands, and increasing numbers of target CpG sites, the kits have advanced from HumanMethylation 27K BeadChip (27K array) to HumanMethylation 450K (450K array) and more recently the Infinium MethylationEPIC (850K array). The following reviews describe the steps involved in data acquisition and processing.

- Review of processing and analysis methods for DNA methylation array data.^253^
- Computational and statistical analysis of array-based DNA methylation data.^254^

In a multicenter study based in Europe, investigators have successfully identified and validated a DNA methylation signature, termed EPIMMUNE, associated with clinical benefit from anti-PD-1 blockade in patients with stage IV lung cancer. Among the methylated genes, forkhead box P1 (FOXP1) was confirmed to be a predictor of clinical benefit from anti-PD-1 therapies. The epigenetic signature was not associated with the PD-L1 status, mutational load, or CD8 immunostaining.

- Epigenetic prediction of response to anti-PD-1 treatment in non-small-cell lung cancer: a multicentre, retrospective analysis.^255^

When compared with studies that require live cells for immune subset quantification and monitoring, assessing gene methylation by epigenetic assays to identify cell subsets is a more feasible approach. The same principle can be used to characterize the cell population of tumor tissue as well.

- Novel technologies and emerging biomarkers for personalized cancer immunotherapy.^7^

PBMCs and T cells from patients with HCC have been shown to have a DNA methylation signature distinct from those who do not have HCC. This methylation signature in the host immune cells deepens as the HCC advances.

- The signature of liver cancer in immune cells DNA methylation.^256^

#### microRNA arrays

Overwhelming portions of the human DNA do not encode protein. The RNA resulting from the transcription of approximately 98% of DNA is non-coding RNA (ncRNA). In the early 2000s, the role of ncRNAs as they affect translation of proteins started to emerge. Further study revealed a large network of interactions between ncRNA and the cell physiological machinery. This has led to a new understanding of what used to be considered ‘junk’ DNA and RNA. These are now recognized as important players in the regulation of the cell machinery and as oncogenic drivers and suppressors.

- Non-coding RNA networks in cancer.^257^

microRNA (miRNA) are 22-nucleotide short RNA molecules that are highly conserved through evolution. miRNA binds to mRNA transcripts of a gene, which leads to cleavage of the mRNA or shutdown of gene translation. miRNAs are active participants in normal cellular, developmental, and host environment processes such as intercellular communication through exosomes. Additionally, these tiny molecules play a fascinating role in cancer, as they have the powerful ability to orchestrate carcinogenesis and therapy resistance in cancer. Some miRNAs may stimulate an oncogenic process directly by acting as a ligand, termed onco-miRNAs (such as miR-21/miR-29a), while others such as miR34a act as tumor suppressors. For a detailed review on this subject, refer to the following.

- The role of microRNAs in human cancer.^258^

Circulating and exosomal miRNAs can be used as diagnostic biomarkers for diseases such as cancer. The following reviews discuss the process of developing miRNAs as biomarkers, various profiling platforms, sample preparation, and analytical strategies.

- MicroRNA as biomarkers and diagnostics.^259^
- Potential pitfalls in microRNA profiling.^260^

The following reviews outline the role of miRNAs in mediating immune responses by influencing cellular signaling in immune cells, influencing both innate and adaptive immunity.

- MiRNAs: dynamic regulators of immune cell functions in inflammation and cancer.^261^
- Nuclear functions of mammalian microRNAs in gene regulation, immunity and cancer.^262^
- Extracellular RNAs: a secret arm of immune system regulation.^263^
- miRNA regulation of innate immunity.^264^
- MicroRNAs as regulatory elements in immune system logic.^265^

The following is an excellent review of the role of miRNAs in oncogenesis and therapy resistance in melanoma. The authors describe an miRNA signature that affects angiogenic and inflammatory pathways and predicts resistance to both targeted and immunotherapy, highlighting this as a biomarker of resistance and a potential therapeutic target.

- MicroRNA-driven deregulation of cytokine expression helps development of drug resistance in metastatic melanoma.^266^

Studies that successfully sought and identified miRNA biomarker signatures in relation to immunotherapy in a variety of settings are listed below.

- Identification of a microRNA signature in dendritic cell vaccines for cancer immunotherapy.^267^
- Circulating immune cell and microRNA in patients with uveal melanoma developing metastatic disease.^268^

miRNA can also be used as therapeutic targets.

- Sequence-specific knockdown of EWS-FLI1 by targeted, nonviral delivery of small interfering RNA inhibits tumor growth in a murine model of metastatic Ewing’s sarcoma.^269^
- Evidence of RNAi in humans from systemically administered siRNA via targeted nanoparticles.^270^

#### Conclusions

Epigenetic biomarker discovery has an expansive reach in clinical practice for diagnostic, prognostic, and therapeutic purposes. Multiple epigenetic processes such as DNA methylation, histone post-translational changes, chromatin remodeling, and ncRNA production have been described in the context of normal tissue development, cancer, and immunology. Sample processing and preservation, miniaturization, and automation have improved the ability to perform large-scale reproducible epigenome-wide biomarker assays to define the epigenetic phenotype of cells. In conjunction with refined bioinformatics analytical processes, epigenetics has been successfully applied toward biomarker discovery in immunology. A shared knowledge of a growing epigenomic database, developed in conjunction with a much larger and more mature genomic database, facilitates establishment of epigenomic signatures of cell subsets, drug resistance, and other immunological biomarkers. Epigenetic changes can serve as biomarkers of diagnosis and prognosis, and are attractive therapeutic targets.

### Transcriptomic biomarker discovery

#### RNA sequencing

With the advent of NGS, RNA-seq has become mainstream in transcriptome analysis spanning basic and translational research. This methodology enabled sequencing and quantification of the transcriptional portraits of individual cells or thousands of samples, linking cellular and molecular phenotypes. Below is the first article that reported RNA-seq.

- Mapping and quantifying mammalian transcriptomes by RNA-seq.^271^

The following articles provide a high-level overview of RNA-seq, summarizing advantages over existing transcriptomic platforms and challenges. Technological advancements are covered, including improvements in transcription start site mapping, strand-specific measurements, gene fusion detection, small RNA characterization, detection of alternative splicing events, direct RNA sequencing, and approaches that enable profiling of small RNA quantities. Also reviewed are methods and tools developed for preprocessing high-throughput RNA-seq data and the analysis of differential gene expression.

- RNA-seq: a revolutionary tool for transcriptomics.^272^
- RNA sequencing: advances, challenges and opportunities.^273^
- From RNA-seq reads to differential expression results.^274^

These articles outline a historical timeline of transcriptomics, summarize various protocols and computational tools for RNA-seq, discuss the potential clinical utility of transcriptomic approaches, provide a useful toolbox with resources to analyze cancer transcriptomics, outline the lack of appropriate reference standards for validating RNA-seq, and illustrate the overabundance of competing computational tools.

- Cancer transcriptome profiling at the juncture of clinical translation.^275^
- Translating RNA sequencing into clinical diagnostics: opportunities and challenges.^276^
- Reference standards for next-generation sequencing.^277^

This article provides links to resources (Ensembl Compara, Gencode, Mouse Genomes Project, Mouse Phenome Database, OMIM (Online Mendelian Inheritance in Man), Rfam (RNA families database), Blueprint, ENCODE (Encyclopedia of DNA Elements), FANTOM (Functional Annotation of the Mammalian Genome), GTEx (Genotype-Tissue Expression) project, Human Cell Atlas Consortium, and so on) to help interrogate human and mouse transcriptomics.

- Comparative transcriptomics in human and mouse.^278^

#### EdgeSeq

EdgeSeq is a gene expression analysis platform developed by HTG which combines quantitative nuclease protection assay technology with NGS, using small amounts of starting material and delivering reproducible GEPs from poor-quality formalin-fixed paraffin-embedded (FFPE) tissue samples including haematoxylin and eosin (H&E)-stained tumor specimens. The EdgeSeq platform is capable of generating reliable expression data for thousands of genes from as little as 1 mm^2^ FFPE tissue and crude FFPE tissue lysates equivalent to surface areas as low as 0.31 mm^2^ of a 5 mm section, and can be particularly useful to interrogate biomarkers in oncology clinical trials, which often lack a sufficient amount of high-quality tumor tissue for other techniques.

- Reliable gene expression profiling from small and hematoxylin and eosin-stained clinical formalin-fixed, paraffin-embedded specimens using the HTG EdgeSeq platform.^279^
- EMT- and stroma-related gene expression and resistance to PD-1 blockade in urothelial cancer.^168^

#### Conclusions

RNA-seq is an open platform technology that has a number of potential advantages over gene expression microarrays, including an increased dynamic range of expression, measurement of focal changes (such as single nucleotide variants, insertions, and deletions), detection of different transcript isoforms, splice variants, and chimeric gene fusions (including previously unidentified transcripts and/or RNA species such as circular RNAs), and application to samples obtained from any biological species. As the cost of RNA-seq continues to decrease, this platform will most likely replace many applications focused on the analysis of transcriptome structure and dynamics. RNA-seq-based assays also have the potential to become a diagnostic platform in different therapeutic areas, including oncology. However, the establishment of appropriate quality standards and the adoption of best practices will be necessary to transform this exciting technology from a purely exploratory tool into a reliable diagnostic platform.

### Single-cell gene expression analysis

The application of RNA-seq to single cells was first published here.

- mRNA-seq whole-transcriptome analysis of a single cell.^280^

The following two articles provide an overview of scRNA-seq applications in immunology; the second paper contains links to useful databases such as the Differentiation Map (DMAP) project, Hematopoietic Stem and Progenitor Cell Atlas, Illumina Body Map Expression Atlas, Immunological Genome Project, and Portal for multiple scRNA-seq data.

- A single-cell sequencing guide for immunologists.^281^
- Single-cell RNA sequencing to explore immune cell heterogeneity.^282^

scRNA-seq has the potential to identify rare immune cell subsets as well as unique cell populations and transcriptomic signatures associated with response or resistance to immunotherapy in humans and mice.

- Single-cell transcriptomics in cancer immunobiology: the future of precision oncology.^283^
- Single-cell RNA-seq reveals new types of human blood dendritic cells, monocytes, and progenitors.^284^
- A cancer cell program promotes T cell exclusion and resistance to checkpoint blockade.^173^
- Defining T cell states associated with response to checkpoint immunotherapy in melanoma.^150^
- High-dimensional analysis delineates myeloid and lymphoid compartment remodeling during successful immune-checkpoint cancer therapy.^285^

These articles provide an overview of currently available scRNA-seq methods and describe methods for the isolation of individual cells for scRNA-seq, construction of cDNA libraries, and computational analysis. They also discuss current applications and challenges associated with scRNA-seq.

- Single-cell RNA-seq: advances and future challenges^286^
- The technology and biology of single-cell RNA sequencing.^287^
- Design and analysis of single-cell sequencing experiments.^288^

A number of biological and technical factors should be taken into consideration to measure the transcriptomic profiles of single cells, and computational methods can be developed to remove technical effects and dissect factors underlying biological variation.

- Revealing the vectors of cellular identity with single-cell genomics.^289^
- Exponential scaling of single-cell RNA-seq in the past decade.^290^
- Computational and analytical challenges in single-cell transcriptomics.^291^
- Design and computational analysis of single-cell RNA-sequencing experiments.^292^
- Challenges in unsupervised clustering of single-cell RNA-seq data.^293^

Clustered regularly interspaced short palindromic repeats (CRISPR)-based genetic screens have been increasingly used in basic research and drug discovery; their use, however, has been restricted to the analysis of simple cellular phenotypes in bulk cell populations. Multiple screening strategies are currently focused on combining CRISPR-based gene alterations with scRNA-seq to enable high-content molecular analysis with single-cell resolution, including cell lineage tracing.

- Dissecting immune circuits by linking CRISPR-pooled screens with single-cell RNA-seq.^294^
- Perturb-Seq: dissecting molecular circuits with scalable single-cell RNA profiling of pooled genetic screens.^295^
- A multiplexed single-cell CRISPR screening platform enables systematic dissection of the unfolded protein response.^296^
- Whole-organism clone tracing using single-cell sequencing.^297^
- Simultaneous lineage tracing and cell-type identification using CRISPR-Cas9-induced genetic scars.^298^
- Simultaneous single-cell profiling of lineages and cell types in the vertebrate brain.^299^

Multimodal data are generated by single-cell transcriptomics, genomics, epigenomics, and proteomics methods to enable integrative analyses. Emerging technologies also allow spatial single-cell gene expression analysis by RNA in situ or other methods such as STARmap, SpatialDE, or trendsceek to allow assessment of the spatial organization of individual cells within a tissue. STARmap is a combination of hydrogel-tissue chemistry, targeted signal amplification, and in situ sequencing which enables mRNA quantification in single cells and positional mapping of cell types. SpatialDE and trendsceek employ statistical methods from geostatistics, astronomy, and materials physics to develop clustering approaches that enable spatial gene expression analysis.

- Integrative single-cell analysis.^300^
- Spatially resolved transcriptomics and beyond.^301^
- Three-dimensional intact-tissue sequencing of single-cell transcriptional states.^302^
- SpatialDE: identification of spatially variable genes.^303^
- Identification of spatial expression trends in single-cell gene expression data.^304^

#### Conclusions

Single-cell transcriptomic analysis is rapidly transforming the field of biomedical research. While the promise and potential of this technology are apparent, several challenges remain. Efforts are underway to improve single-cell partitioning and whole transcriptome amplification, and to increase the sensitivity of scRNA-seq, which would allow detection of low-abundance RNAs and rare cells in the presence of biological and technical noise. An appropriate sample size and measurement of sufficient numbers of single-cell events are equally important to increase the accuracy and precision of scRNA-seq analyses. There is also a need for standardized scRNA-seq protocols, harmonized computational pipelines (including methods capable of resolving spatial single-cell transcriptomics), integrated single-cell data across experiments or modalities, and repositories dedicated to the massive and constantly increasing amounts of scRNA-seq data.

### Hybridization and PCR-based gene expression platforms

#### Transcriptome profiling with microarrays

While commonly used DNA arrays have become quickly outdated with the advent of RNA-seq, more advanced gene expression microarrays (eg, Affymetrix Human Transcriptome Array V.2.0 (HTA V.2.0), Clariom D and S arrays), which leverage the latest transcriptome data from multiple databases, are simple and fast tools for whole-transcriptome expression profiling and biomarker discovery. The Clariom D array is based on differential exon usage resulting from alternative splicing; this platform may be advantageous for the assessment of millions of distinct sequences and could be particularly useful in detecting and quantifying low abundance transcripts, or rare alternative splice variants. It is also worth noting that Clariom D arrays require very low RNA input and are compatible with formalin-fixed biological samples.

- https://www.thermofisher.com/us/en/home/life-science/microarray-analysis/transcriptome-profiling-microarrays/arrays-rna-seq.html^305^

HTA V.2.0 was used for gene expression profiling to allow analysis of coding as well as non-coding and alternatively spliced transcripts in peripheral T cells isolated from patients with melanoma treated with anti-PD-1, anti-CTLA-4, and combinations of both antibodies. The results of this analysis revealed quantitatively and qualitatively distinct gene expression signatures associated with monotherapy or combination treatment.

- Combination therapy with anti-CTLA-4 and anti-PD-1 leads to distinct immunologic changes in vivo.^176^

#### nCounter and Digital Spatial Profiler

The nCounter gene expression system developed by NanoString enables enumeration of individual mRNAs using unique barcoding technology. Advantages over existing platforms include direct measurement of mRNA expression levels without enzymatic reactions, sensitivity coupled with high multiplex capability (up to 800 transcripts), and digital readout. Because detection probes in the nCounter analysis target relatively short mRNA sequences, this platform demonstrates outstanding performance in FFPE tissue samples and can be run on both purified RNA and tissue/cell lysates. It has also demonstrated utility in preclinical research using different species (mouse, rhesus/cynomolgus monkey) and sample types (tumor tissue, whole blood).

- Direct multiplexed measurement of gene expression with color-coded probe pairs.^306^
- The CDK4/6 inhibitor abemaciclib induces a T cell inflamed tumor microenvironment and enhances the efficacy of PD-L1 checkpoint blockade.^307^
- A conserved transcriptional response to intranasal Ebola virus exposure in nonhuman primates prior to onset of fever.^308^
- Evaluating robustness and sensitivity of the NanoString technologies nCounter platform to enable multiplexed gene expression analysis of clinical samples.^309^

The nCounter platform has been used to interrogate predictive and pharmacodynamic biomarkers in immuno-oncology clinical trials.

- IFN-γ-related mRNA profile predicts clinical response to PD-1 blockade.^164^
- Pan-tumor genomic biomarkers for PD-1 checkpoint blockade-based immunotherapy.^118^
- Analysis of immune signatures in longitudinal tumor samples yields insight into biomarkers of response and mechanisms of resistance to immune checkpoint blockade.^143^

Digital Spatial Profiler (DSP) is another platform developed by NanoString aimed at assisting with positional information about mRNA/protein expression in a tissue sample. DSP is essentially a variation of multiplex mRNA in situ hybridization and IHC, but is based on the nCounter barcoding technology. The platform is capable of providing spatially resolved, digital characterization of proteins or mRNA in a highly multiplexed (up to 1000-plex) assay. The following two articles exemplify the translational utility of the DSP assay.

- Neoadjuvant immune checkpoint blockade in high-risk resectable melanoma.^310^
- Neoadjuvant vs adjuvant ipilimumab plus nivolumab in macroscopic stage III melanoma.^311^

#### QuantiGene Plex

The QuantiGene Plex (QGP) gene expression assay combines branched DNA (bDNA) technology with the Luminex fluorescent microbead-based platform. It uses cooperative hybridization, which allows for an exceptionally high degree of assay specificity by using multiple probes that hybridize to the same gene. The QGP assay is amenable to high-throughput analysis in a 96-well or 384-well format and is capable of multiplexing up to 80 targets in one well. Similar to nCounter, this platform is compatible with formalin-fixed material or samples with degraded RNA and can be run on both purified RNA and tissue/cell lysates.

- A multiplex branched DNA assay for parallel quantitative gene expression profiling.^312^
- QuantiGene Plex represents a promising diagnostic tool for cell-of-origin subtyping of diffuse large B-cell lymphoma.^313^
- The CDK4/6 inhibitor abemaciclib induces a T cell inflamed tumor microenvironment and enhances the efficacy of PD-L1 checkpoint blockade.^307^

#### High-throughput quantitative PCR

The microfluidics-based Biomark HD (Fluidigm) is a moderate/high-throughput qPCR system which is capable of analyzing the expression of multiple genes across multiple samples (up to 96×96) on a single plate with an integrated fluidic circuit format. Compared with plate-based high-throughput qPCR platforms, the Biomark HD provides excellent flexibility coupled with time-effectiveness and cost-effectiveness to explore a range of transcriptomic biomarkers in clinical samples. It is capable of analyzing samples with very low RNA input. However, as has been observed with all PCR assays, the Biomark HD demonstrates superior performance on RNA isolated from snap-frozen rather than formalin-fixed tissue samples.

The below referenced articles provide examples of high-throughput qPCR analysis of formalin-fixed tumor biopsy samples using Biomark HD as part of biomarker assessments in clinical trials of anti-PD-L1 (atezolizumab).

- Predictive correlates of response to the anti-PD-L1 antibody MPDL3280A in cancer patients.^87^
- Atezolizumab versus docetaxel for patients with previously treated non-small-cell lung cancer (POPLAR): a multicentre, open-label, phase 2 randomised controlled trial.^33^

#### Conclusions

Although RNA-seq has been increasingly used in biomedical research and may be advantageous in biomarker discovery as an agnostic hypothesis-generating tool, under certain circumstances the aforementioned gene expression platforms may provide significant value, particularly in situations when limited amounts of tissue material or formalin-fixed tissue samples are available. While exon junction (Clariom D) arrays and nCounter assays allow broad interrogation of transcriptomic changes, QGP and high-throughput-qPCR assays are more fit for hypothesis testing or interrogation of specific transcriptomic biomarkers or signatures.

### Proteomic biomarkers discovery: detection techniques

#### ELISPOT

ELISPOT allows the detection of functionally active, antigen-specific immune cells on the single cell level by capturing the released analyte of interest on a membrane, which is then made visible for enumeration. The assay format as used today for the detection of cytokine-secreting cells was first described in 1988.

- Reverse ELISPOT assay for clonal analysis of cytokine production. I. Enumeration of gamma interferon-secreting cells.^314^

As a generally easy-to-perform assay with exceptional sensitivity, ELISPOT has remained a common assay choice for basic, translational, and clinical applications in a variety of fields, with surprisingly little change to the basic procedure. A comprehensive review of the technique can be found here.

- Elispot for rookies (and experts too), techniques in life science and biomedicine for the non-expert.^315^

ELISPOT has been the subject of broad harmonization efforts.

- Results and harmonization guidelines from two large-scale international Elispot proficiency panels conducted by the Cancer Vaccine Consortium (CVC/SVI).^316^
- Guidelines for the automated evaluation of Elispot assays.^317^

With the introduction of fluorophores for spot detection (FluoroSpot), multiplexing is now possible and is currently being used for the polyfunctional assessment of cells for up to four different cytokines, resulting in the potential detection of 15 subpopulations. The application of peptide-tagged antigen and anti-tag detection antibodies can be used to identify multiple antibody-secreting cells with different antigen specificities.

- An antigen-specific, four-color, B-cell FluoroSpot assay utilizing tagged antigens for detection.^318^

While the analysis of ELISPOT and FluoroSpot has largely depended on image analyzers, a new analysis algorithm uses data directly from the camera chip, avoiding any evaluation bias and providing spot volume data, a relative measure of the amount of cytokine released by a single cell.

- Cell detection by functional inverse diffusion and non-negative group sparsity—part I-II.^319^

An ELISPOT-specific statistical test for response definition has been developed, and an online tool is available to the community for free.

- http://www.scharp.org/zoe/runDFR/320

Many publications exist using ELISPOT for immune monitoring purposes in immunotherapeutic cancer trials, including the neoantigen arena.

- An immunogenic personal neoantigen vaccine for patients with melanoma.^321^

The monitoring of antigen-specific immune responses in patients undergoing immunotherapy can be performed using ex vivo isolated T cells without the bias of in vitro stimulation.

- Immunological markers and clinical outcome of advanced melanoma patients receiving ipilimumab plus fotemustine in the NIBIT-M1 study.^322^

##### Conclusions

ELISPOT and FluoroSpot are useful tools for functionally assessing immune cells at the single cell level, and also allow the polyfunctional analysis of cells. It is, however, generally not possible to do phenotypic analysis of cells with these methods.

#### ELISA

The Enzyme Linked Immunosorbent Assay (ELISA) is a method first described by Weiland^323^ in 1978 to detect and quantify the presence of analytes, including antibodies, antigens, proteins, and glycoproteins in biological samples.

- The enzyme-linked immunosorbent assay (ELISA)--a new serodiagnostic method for the detection of parasitic infections.^323^

This method has been widely used for routine diagnosis of viral diseases, such as HIV or HBV infection, but also for pregnancy tests and quantification of soluble molecules in patients’ serum, plasma, urine, or cellular supernatants. ELISAs can be performed in 48-well, 96-well, and 384-well plates, allowing concomitant interrogation of multiple samples, as well as monitoring of changes in analyte concentration at different timepoints.

The most common types of ELISA are the (1) indirect and (2) sandwich methods. The indirect ELISA is generally used to detect antibodies in the serum, plasma, or supernatants. Cancer patients’ humoral immune responses against specific tumor-associated antigens (TAAs) have been assessed with the aim of monitoring changes in immune responses over the course of treatments and to find possible associations with clinical outcome. Humoral responses against shared TAAs, such as NY-ESO-1, p53, and SOX2, have been monitored in serum or plasma of patients with cancer using ELISAs.

- Autoantibodies against cancer antigens.^324^
- Integrated NY-ESO-1 antibody and CD8^+^ T cell responses correlate with clinical benefit in advanced melanoma patients treated with ipilimumab.^325^
- Ipilimumab increases activated T cells and enhances humoral immunity in patients with advanced melanoma.^326^
- Mechanistic insight into the TH1-biased immune response to recombinant subunit vaccines delivered by probiotic bacteria-derived outer membrane vesicles.^327^

The sandwich method allows the detection of soluble antigens. In this technique, an analyte-specific antibody is coated on the microtiter well. The sample to be analyzed is then added to the well, forming an antigen-antibody complex. A second enzyme-conjugated antibody specific for a different epitope on the antigen is added and, in the presence of an enzyme-specific substrate, the colorimetric reaction is developed. This method allows detection of soluble factors in serum or plasma, including cytokines, immunomodulating molecules, and growth factors, as well as their changes in association with the clinical outcome of patients with cancer undergoing immunotherapy treatments.

- Contribution of humoral immune responses to the antitumor effects mediated by anthracyclines.^328^
- Soluble NKG2D ligands are biomarkers associated with the clinical outcome to immune checkpoint blockade therapy of metastatic melanoma patients.^329^
- Immunological markers and clinical outcome of advanced melanoma patients receiving ipilimumab plus fotemustine in the NIBIT-M1 study.^322^
- A pilot phase I study combining peptide-based vaccination and NGR-hTNF vessel targeting therapy in metastatic melanoma.^330^

The sensitivity of ELISA can be augmented through the amplified luminescent proximity homogeneous assay (ALPHA). This chemiluminescence-based method can detect analytes at the level of femtograms, reduces washing steps, and is based on the usage of acceptor beads coated with the primary antibody specific for the defined antigen, streptavidin-coated donor beads, and the secondary antibody conjugated to streptavidin.

##### Conclusions

ELISA is a simple methodology that provides rapid results. No antigen purification is required prior to measurement and specificity is increased by using two antibodies. Flexibility and sensitivity are increased by the application of ALPHA technology, allowing the usage of a minimal amount (5–20 µL) of starting material and large-scale screening (96-well, 384-well, or 1536-well format). However, these methods are limited by the detection of one analyte at a time and do not allow multiplex high-throughput screening.

#### Multiplexed immunoassays

In the late 1990s, the recently founded Luminex Corporation proposed a commercial FlowMetrix platform for the simultaneous detection of up to 64 analytes using a conventional flow cytometer. The technology relied on 64 distinct sets of fluorescent beads, each coupled with either an antigen, or an antibody, or a nucleic acid, and used as the solid phase of immunoassays or hybridization. The mixing of these distinct sets allowed for the simultaneous monitoring of independent assays using a flow cytometer equipped with digital signal processing.

- Advanced multiplexed analysis with the FlowMetrix system.^331^

This seminal publication laid the basis of the future development of high dimension protein and nucleic acid profiling platforms. In its current implementation, the Luminex platform is able to monitor 500 simultaneous assays. The versatility of the platform has given rise to several applications in cancer research. A comprehensive review of the origin and evolution of multiplex assays has recently been discussed by Graham H *et al.*^332^

- The genesis and evolution of bead-based multiplexing.^322^

The simplest application consists of the evaluation of cytokine and chemokine profiles in the serum or plasma of patients affected by cancers.

- The plasma levels of 12 cytokines and growth factors in patients with gastric cancer.^333^
- Cytokine comparisons between women with breast cancer and women with a negative breast biopsy.^334^

Luminex assays or other multiplexed bead array embodiments, such as the Cytokine Bead Array (Becton Dickinson), performed on peripheral blood or other body fluids, have been used as prognostic/diagnostic tools in several cancers.

##### Ovarian cancer

- Serum cytokine profiling as a diagnostic and prognostic tool in ovarian cancer: a potential role for interleukin 7.^335^
- Diagnostic markers for early detection of ovarian cancer.^336^
- Multiplexed bead-based immunoassay of four serum biomarkers for diagnosis of ovarian cancer.^337^
- Serum expression level of cytokine and chemokine correlates with progression of human ovarian cancer.^338^

##### Breast cancer

- The multiplex bead array approach to identifying serum biomarkers associated with breast cancer.^339^

##### Nasopharyngeal carcinoma

- Prognostic role of serum cytokines in patients with nasopharyngeal carcinoma.^340^

##### Gastric cancer

- Serum biomarker panels for diagnosis of gastric cancer.^341^

##### Colorectal cancer

- Diagnostic performance of a novel multiplex immunoassay in colorectal cancer.^342^

##### Non-Hodgkin lymphoma

- Cytokines in serum in relation to future non-Hodgkin lymphoma risk: evidence for associations by histologic subtype.^343^

##### Non-small cell lung carcinoma

- A novel detection method of non-small cell lung cancer using multiplexed bead-based serum biomarker profiling.^344^
- Determination of 16 serum angiogenic factors in stage I non-small cell lung cancer using a bead-based multiplex immunoassay.^345^
- Salivary cytokine panel indicative of non-small cell lung cancer.^346^
- Evaluation of saliva and plasma cytokine biomarkers in patients with oral squamous cell carcinoma.^347^

Multiplexed immunoassays have entered the arena of personalized medicine to monitor response to therapy in patients affected by NSCLC.

- Differential expression of circulating biomarkers of tumor phenotype and outcomes in previously treated non-small cell lung cancer patients receiving erlotinib vs cytotoxic chemotherapy.^348^

In addition to biomarker discovery, multiplexed bead assays have been used to characterize the phosphorylation pathways whose dysregulation is often involved in carcinogenesis. A technical chapter describing this approach can be found in the following.

- Utilizing the Luminex magnetic bead-based suspension array for rapid multiplexed phosphoprotein quantification.^349^

The ability of Luminex assays to monitor DNA hybridization has been exploited to characterize mutations in cancer cells.

- Clinical validation of newly developed multiplex kit using Luminex xMAP technology for detecting simultaneous RAS and BRAF mutations in colorectal cancer: results of the RASKET-B study.^350^

Bead multiplexing and PCR multiplexing have been combined in multiplex liquid bead arrays used for the molecular characterization of circulating tumor cells (CTCs).

- Molecular characterization of circulating tumor cells in breast cancer by a liquid bead array hybridization assay.^351^
- Development and validation of multiplex liquid bead array assay for the simultaneous expression of 14 genes in circulating tumor cells.^352^

Another multiplex immunoassay platform, Olink, uses proximity extension assay (PEA) technology to achieve specificity for 92 analytes in a single panel, with multiple disease-specific panels available. PEA uses a pair of non-cross-blocking antibodies for each target, which are tagged with complementary oligonucleotides. Binding of the antibody pair allows for the complementary sequences to pair and to be extended to create a PCR template. Readout is on the Fluidigm Biomark microfluidic qPCR platform, using a 96.96 array (96 assay targets × 96 samples/controls). Because of the availability of an immune oncology panel, Olink is being used with increasing frequency in immunotherapy trial monitoring, including in the CIMAC/CIDC network. For example, this CAR T cell study used Olink to find multiple plasma proteins that correlated with responder status, including IL-12.

- A phase I/IIa trial using CD19-targeted third-generation CAR T cells for lymphoma and leukemia.^353^

##### Conclusions

Multiplexed bead arrays are a useful tool in cancer research, with applications ranging from diagnosis, disease monitoring, predictive and prognostic biomarkers, to the molecular characterization of cancer cells at the transcriptome and protein levels.

### Cytometry-based methods

Flow cytometry remains a very powerful tool for multiparameter analysis of immune cells in both blood and tumor tissue. This section highlights advances in flow cytometry-related methodology that impacts the field of immunotherapy.

#### High-parameter flow cytometry

The number of parameters that can be measured in parallel by multicolor flow cytometry has grown rapidly in the last 8 years, mostly due to the development of new polymer-based dyes with tunable emission peaks, as described in the following.

- Brilliant violet fluorophores: a new class of ultrabright fluorescent compounds for immunofluorescence experiments.^354^

At the same time, new instrumentation has now made it possible to perform flow cytometry with over 18 colors (the previous limit), the limiting factor currently being the fluorochromes. This has allowed the simultaneous monitoring of a large number of immune checkpoint molecules on a wide array of immune cell types, as shown with this 28-color panel.

- OMIP-050: A 28-color/30-parameter fluorescence flow cytometry panel to enumerate and characterize cells expressing a wide array of immune checkpoint molecules.^355^

Another notable development in the field is flow cytometers that use spectral deconvolution rather than a single bandpass filter/detector for each fluorochrome. In effect, spectral cytometry uses the shape of the complete emission spectrum to determine the signals derived from each fluorochrome on each cell. This has allowed resolution of fluorochromes that would otherwise be very difficult to distinguish. A review of how this method has been applied to cells derived from solid tissues, a common application for tumor immune monitoring, is given in the following.

- Spectral cytometry has unique properties allowing multicolor analysis of cell suspensions isolated from solid tissues.^356^

The use of standardized multicolor immune monitoring panels to profile immune cell phenotypes and functions in blood and tumor is exemplified in this recent publication in breast cancer.

- Examining peripheral and tumor cellular immunome in patients with cancer.^357^

##### Conclusions

Fluorescence-based flow cytometry continues to evolve, with new dyes and new instrumentation, including spectral flow cytometers that increase the number of parallel parameters that can be measured, as well as the resolution of those parameters.

#### Mass cytometry

Flow cytometry is performed using fluorescently tagged antibodies and other fluorescent probes. More recently, the use of heavy metal ion tags, which are chelated to a polymer backbone and read out using time-of-flight mass spectrometry, has been introduced. This method is known as mass cytometry, or CyTOF (Cytometry by Time of Flight). While offering a significantly slower throughput than fluorescence cytometry, it provides the dual advantages of allowing for more antibody specificities to be used in parallel, along with much reduced spillover between detector channels. The method was first described in the following papers.

- Development of analytical methods for multiplex bio-assay with inductively coupled plasma mass spectrometry.^358^
- Flow cytometer with mass spectrometer detection for massively multiplexed single-cell biomarker assay.^359^

CyTOF technology was first applied to the study of the immune system in 2010, using a panel of 32 antibodies on human bone marrow cells.

- Single-cell mass cytometry of differential immune and drug responses across a human hematopoietic continuum.^360^

The number of available mass tags has since grown to over 40, with many publications in different disease settings. It has been used to study signaling in leukemic cells, as well as immune signatures in PBMCs in the context of immunotherapy, as in the following representative publications.

- Mass cytometric functional profiling of acute myeloid leukemia defines cell-cycle and immunophenotypic properties that correlate with known responses to therapy.^361^
- Data-driven phenotypic dissection of AML reveals progenitor-like cells that correlate with prognosis.^362^
- Distinct predictive biomarker candidates for response to anti-CTLA-4 and anti-PD-1 immunotherapy in melanoma patients.^54^

Mass cytometry has also been applied to the study of immune cells infiltrating tumor tissue, as in the following recent publications.

- Interlesional diversity of T cell receptors in melanoma with immune checkpoints enriched in tissue-resident memory T cells.^363^
- An immune atlas of clear cell renal cell carcinoma.^364^

A review of technology and important concepts for CyTOF data analysis has been recently published.

- The anatomy of single cell mass cytometry data.^365^

The above review also links to a website containing a tutorial on mass cytometry data analysis and various tools for the same.

- http://cytof.biosurf.org/366

A recent study has made available an ‘Antibody Staining Data Set’ which shows the estimated expression level of 326 cell-surface proteins on 28 different immune cell types, on fresh or fixed cells. The interactive heat map tool can be viewed at the following.

- https://app.astrolabediagnostics.com/antibody_staining_data_set%23:%7E:text=The_Antibody_Staining_Data_Set,subsets_at_single-cell_resolution^367^

##### Conclusions

Mass cytometry, while posing some technical challenges, allows for the use of more antibody specificities than fluorescence flow cytometry, and with much less spillover between detector channels. It is well suited to comprehensive analyses of immune cells in tissues such as blood, lymph nodes, or tumor. However, see multiplexed ion beam imaging in the ‘Mass spectrometry for tissue multiplexing’ section for an alternative approach to highly multiplexed analysis of tumor tissue, which preserves the spatial orientation of the tissue cells.

#### Multimers

Multimeric forms of MHC–peptide complexes can be identified to stain T cells specific for a given MHC–peptide combination. This was first demonstrated using tetrameric forms of MHC–peptide, bound together using fluorescently labeled streptavidin.

- Phenotypic analysis of antigen-specific T lymphocytes.^368^

From this work, the term ‘tetramer’ emerged as a common name for these reagents. However, other versions of peptide–MHC multimers have also been made, with backbones other than streptavidin, and with differing resulting valencies. The timeline of development of various MHC multimer reagents is reviewed here.

- Interrogating the repertoire: broadening the scope of peptide-MHC multimer analysis.^369^

More recent efforts have focused on the parallel use of many different MHC–peptide multimers, which is facilitated by combinatorial staining of each multimer species using a different ‘barcode’ or combination of fluorescent labels.

- Simultaneous detection of many T-cell specificities using combinatorial tetramer staining.^370^
- Parallel detection of antigen-specific T-cell responses by multidimensional encoding of MHC multimers.^371^

Such parallelization has also been accomplished using heavy metal-tagged tetramers and mass cytometry.

- Combinatorial tetramer staining and mass cytometry analysis facilitate T-cell epitope mapping and characterization.^372^
- Multiplexed peptide-MHC tetramer staining with mass cytometry.^373^

Application of barcoded tetramers, along with nanoparticle display to increase avidity, has been applied to the tracking of neoantigen-specific T cells in tumors and blood.

- Sensitive detection and analysis of neoantigen-specific T cell populations from tumors and blood.^374^

To create many different MHC–peptide multimers for a given MHC protein, one can use a peptide-exchange approach. With this method, a single MHC protein is produced with an invariant peptide in its binding groove. This is then exchanged for any peptide of interest, as described here.

- Design and use of conditional MHC class I ligands.^375^

Arrays of MHC–peptide multimers can be used to screen heterogeneous T cell populations for binding. This provides a way to determine the specificities of T cells for neoantigens, for example.

- High throughput determination of the antigen specificities of T cell receptors in single cells.^376^

##### Conclusions

The use of MHC–peptide multimers to identify T cell specificities is limited by the fact that each multimer reagent identifies T cells specific for a single epitope in the context of a single MHC allele. Historically, this has restricted their use in tumor immune monitoring to settings such as peptide vaccination, where the patient’s human leukocyte antigen (HLA) type is selected, and there is only one or a few specificities of interest. More recently, the specificity limitation has been partly overcome by the use of many multimer reagents in parallel; these reagents can be barcoded with unique combinations of fluorescent or mass labels, to minimize the number of detection channels required. The production of many multimers using the same MHC protein is in turn facilitated by a peptide exchange manufacturing approach. Once created, an array of peptide–MHC multimers can be used to efficiently screen patient T cells for their specificity to different neoantigen epitopes.

#### Phospho-flow

The use of antibodies to specific phosphoepitopes, in combination with flow cytometry phenotyping, was pioneered in the lab of Garry Nolan, and has been called ‘phospho-flow’. The earliest papers from the Nolan lab described the use of this technique to profile intracellular signaling in either immune or cancer cells.

- Intracellular phospho-protein staining techniques for flow cytometry: monitoring single cell signaling events.^377^
- Single cell profiling of potentiated phospho-protein networks in cancer cells.^378^
- Multiparameter analysis of intracellular phosphoepitopes in immunophenotyped cell populations by flow cytometry.^379^

##### Conclusions

Identification of signaling anomalies by phospho-flow can be useful for probing both tumor and immune cells. See the ‘Mass cytometry’ section for recent studies using CyTOF-based phospho-flow to distinguish outcome groups in acute myeloid leukemia based on signaling properties.

#### AbSeq/CITE-seq

In order to analyze the binding of many antibody specificities in parallel on single cells, high-throughput sequencing-based approaches have been developed. These methods use nucleic acid-tagged antibodies and barcoding of single cells with indexing beads, which capture the nucleic acid tags from each cell for high-throughput sequencing. This approach, dubbed AbSeq (antibody-based sequencing), was first published in 2017.

- Abseq: Ultrahigh-throughput single cell protein profiling with droplet microfluidic barcoding.^380^

It is possible to combine AbSeq with transcriptomic profiling, a method termed cellular indexing of transcriptomes and epitopes by sequencing (CITE-seq) or RNA expression and protein sequencing (REAP-seq).

- Simultaneous epitope and transcriptome measurement in single cells.^381^
- Multiplexed quantification of proteins and transcripts in single cells.^382^

A protocol for performing such combined proteomic and transcriptomic profiling on a microchip-based single-cell genomics platform has been recently published.

- Protein- and sequencing-based massively parallel single-cell approaches to gene expression profiling.^383^

##### Conclusions

AbSeq has the potential to profile many more antibody specificities in parallel than either fluorescence or mass cytometry. In theory, the number of antibodies used is limited only by cross-blocking of epitopes. However, it is still an expensive method, and is generally performed on a few thousand, but not millions, of cells per sample. It is also not well suited to the study of intracellular proteins, because fixation interferes with the ability to sequence the nucleic acid tags. Combining AbSeq with transcriptomic analysis, for example, CITE-seq, provides a novel way to assess both RNA and protein expression in a highly multiplexed manner on single cells. It may be especially well suited to extracting maximal information about immune cells infiltrating a tumor.

#### Real-time functional assays

The xCELLigence technology (Acea Biosciences) has been developed for cell-based electric impedance assays to monitor and quantify cell proliferation, toxicity, and morphology changes. This platform allows the monitoring of changes in the behavior and adhesion properties of cells through the measurement of impedance, using gold-plated base biosensors in the bottom of culture wells. Cellular status is monitored by label-free and real-time automated reading.

This application can assess cell viability perturbations and determine either cell toxicity and cell death or modification of cell proliferation on treatment with compounds or drugs. Its real-time acquisition of data is suitable to identify the optimal timepoints for a defined cellular activity and to understand the related mechanisms. The xCELLigence technology has increasingly been applied to immunotherapy in order to better understand the complex interaction of immune cells with tumor cells and to verify the efficacy of different immunotherapy approaches or their combinations. Examples are the treatment of cells with either biological agents (eg, agonist or antagonist monoclonal antibodies, or bispecific T cell engagers) or through coculture with effector cells (eg, T, NK, or CAR T cells).

- xCELLigence system for real-time label-free monitoring of growth and viability of cell lines from hematological malignancies.^384^
- Application of real-time cell electronic analysis system in modern pharmaceutical evaluation and analysis.^385^
- In vitro immunotherapy potency assays using real-time cell analysis.^386^
- Avidity characterization of genetically engineered T-cells with novel and established approaches.^387^

##### Conclusions

The xCELLigence platform can provide real-time quantitative determination of functional changes in cells, providing highly reproducible results with a simple workflow. It represents a useful tool to investigate and monitor the mechanisms of action of immunotherapy approaches. This method allows the monitoring of the functional properties of immune cells and other biological drugs, although no extensive results are available regarding its usage to either predict or correlate with the in vivo activity of these therapeutic tools. The combination of xCELLigence with highly dimensional immune phenotype profiling is warranted to achieve deep characterization of immune responses associated with immunotherapy interventions.

### Proteomic biomarkers discovery: target identification and immunomonitoring

#### Minimum residual disease detection

Highly sensitive, standardized techniques are necessary for the detection of minimal residual disease (MRD) in order to assess therapy responses and provide more accurate prognoses, leading to improved personalized treatments. Molecular techniques, such as allele-specific oligonucleotide quantitative PCR (ASO-qPCR), represent the traditional approaches.

- High applicability of ASO-RQPCR for detection of minimal residual disease in multiple myeloma by entirely patient-specific primers/probes.^388^

A major drawback of ASO-qPCR, namely the necessity of using patient-specific probes for B cell malignancies exhibiting somatic hypermutation (eg, multiple myeloma (MM)), is overcome by NGS (also known as deep sequencing or high-throughput sequencing) of immunoglobulin genes.

- Prognostic value of deep sequencing method for minimal residual disease detection in multiple myeloma.^389^
- A clinical perspective on immunoglobulin heavy chain clonal heterogeneity in B cell acute lymphoblastic leukemia.^390^

Flow cytometry classically provided lower sensitivity than molecular techniques. However, next-generation flow cytometry (NGF) approaches reach sensitivities comparable with molecular methods, and have the advantage of faster turnaround times, broader applicability, and lower cost.

- Next generation flow for highly sensitive and standardized detection of minimal residual disease in multiple myeloma.^391^
- ClonoSEQ assay for the detection of lymphoid malignancies.^392^

While MRD detection is an established tool in the management of hematological malignancies, highly sensitive liquid biopsy assays for the assessment of disseminated tumor cells in the context of solid tumors have been developed more recently. These probe for CTCs or circulating tumor-derived factors such as ctDNA, as for example, Natera’s Signatera assay, which provides early disease recurrence prediction based on ctDNA. Since CTCs are rare events, marker-dependent (such as Menarini’s DEPArray) or marker-independent techniques (eg, microfiltration) are applied to enrich them prior to downstream analysis.

- Analysis of plasma cell-free DNA by ultradeep sequencing in patients with stages I to III colorectal cancer.^393^
- Liquid biopsy and minimal residual disease – latest advances and implications for cure.^394^

These techniques as well as additional techniques such as droplet digital PCR (ddPCR), positron emission tomography (PET)-CT, PET-MRI, CyTOF, and monoclonal immunoglobulin rapid accurate mass measurement (miRAMM) are being explored as complementary or alternative approaches for MRD assessment as discussed here.

- Droplet digital PCR for minimal residual disease detection in mature lymphoproliferative disorders.^395^
- Minimal residual disease in multiple myeloma: impact on response assessment, prognosis and tumor heterogeneity.^396^

##### Conclusions

Detection and monitoring of MRD play an important role in the management of patients with hematological malignancies. The somatic hypermutation seen in MM means that many molecular approaches, although exhibiting high sensitivities, are not applicable to all patients, and that false-negatives remain a challenge. With the advent of standardized NGF that is applicable to all patients and reaches comparable sensitivities with molecular techniques, turnaround times and costs are vastly reduced. All of these assays rely on blood or bone marrow samples and are therefore prone to sampling errors, as they ignore spatial heterogeneity in clones. Complementing molecular or flow cytometry-based assays with imaging techniques will enrich MRD readouts, improving clinical follow-up. Highly sensitive techniques are also starting to be harnessed for the detection of tumor-derived material or single tumor cells disseminated from primary solid tumor lesions in liquid biopsies.

#### Neoantigens

Neoantigens are potentially immunogenic epitopes that are created by tumor mutations or chromosomal rearrangements in the course of an individual’s cancer development. They have emerged as an important target of antitumor T cells, and this reactivity may be important both in the context of ICIs as well as adoptive cell therapy (ACT).

##### Detection of neoantigens and neoantigen-specific T cells

Robbins PF *et al*^397^ described a method to identify mutated proteins in patient tumors that may be targets of antitumor T cell immunity using whole-exome sequencing data and MHC binding algorithms.

- Mining exomic sequencing data to identify mutated antigens recognized by adoptively transferred tumor-reactive T cells.^397^

In a study by Linnemann C *et al*,^398^ autologous immortalized B lymphoblastoid cell lines and in silico prediction models are used to demonstrate recognition of neoepitopes by CD4^+^ T cells.

- High-throughput epitope discovery reveals frequent recognition of neo-antigens by CD4^+^ T cells in human melanoma.^398^

As mentioned in the ‘Multimers’ section, stable tetramers can be synthesized using ultraviolet-mediated peptide exchange. This method has been applied to identify neoantigen-specific T cells.

- Generation of peptide MHC class I complexes through UV mediated ligand exchange.^399^

Various methods have been used to screen for the targets of TCRs identified in tumor tissues or other settings. These include screening of yeast display libraries bearing peptide-human leukocyte antigen (pHLA), lentiviral delivery of antigen libraries for display by HLA, identification of pHLA that appropriate TCRs from T cells via trogocytosis, and use of signaling and antigen-presenting bifunctional receptors.

- Antigen identification for orphan T cell receptors expressed on tumor-infiltrating lymphocytes.^400^
- T-Scan: a genome-wide method for the systematic discovery of T cell epitopes.^401^
- T cell antigen discovery via trogocytosis.^402^
- T cell antigen discovery via signaling and antigen-presenting bifunctional receptors.^403^

Another approach uses tetramer-associated TCR-seq to link TCR sequences to their cognate antigen in single cells. This approach has the potential advantage of high-throughput evaluation of antigen specificities.

- High throughput determination of the antigen specificities of T cell receptors in single cells.^376^

The evolving dynamic of tumor targeting by neoantigen-specific T cells and resulting escape of tumor cells lacking the neoantigen(s) contributes to ‘immunoediting’, described in this seminal publication.

- Neoantigen landscape dynamics during human melanoma-T cell interactions.^404^

In view of the complexity of cancer genomes, there is a need to explore unbiased approaches to identify the full range of immunogenic peptides presented by tumors (the ‘immunopeptidome’). These strategies have typically relied on mass spectrometry, immunoprecipitation, or peptide elution, for example.

- Predicting immunogenic tumour mutations by combining mass spectrometry and exome sequencing.^405^

In this approach, whole exome and transcriptome data were combined with mass spectrometry and in silico methods to identify immunogenic mutations.

- Antigen presentation profiling reveals recognition of lymphoma immunoglobulin neoantigens.^406^

Another strategy combined exome sequencing with MHC isolation and peptide identification to uncover tumor neoantigens from ovarian carcinoma cell lines.

- The immunopeptidomic landscape of ovarian carcinomas.^407^

This approach uses chemical methods followed by mass spectrometry analysis to identify HLA binding peptides from direct analysis of tumor cells. Candidate antigens are then validated with complementary methods.

- HLA ligandome analysis identifies the underlying specificities of spontaneous antileukemia immune responses in chronic lymphocytic leukemia (CLL).^408^

##### Conclusions

Strategies for unbiased identification of HLA-binding and/or immunogenic peptides from tumor cells have the potential to provide targets for novel vaccine strategies. These have also led to the identification of both mutated as well as non-mutated peptides as targets for immunotherapy.

##### Role of neoantigens in immunotherapy

Please refer to the TMB section for the role of neoantigens in the response to ICIs. In addition, unique or shared neoantigen-directed, genetically engineered cells have been used as a cellular immunotherapy and have led to regression of tumors.

- Cancer immunotherapy based on mutation-specific CD4^+^ T cells in a patient with epithelial cancer.^409^
- T-cell transfer therapy targeting mutant KRAS in cancer.^410^
- Mutated nucleophosmin 1 as immunotherapy target in acute myeloid leukemia.^411^
- Neoantigen screening identifies broad TP53 mutant immunogenicity in patients with epithelial cancers.^412^

##### Conclusions

In view of the growing importance of neoantigen-specific T cells in tumor immunity, several approaches to identify and expand these cells have been developed. These strategies also demonstrate the feasibility of harnessing these T cells for cancer therapy.

#### Proliferation and cytotoxicity assays

Ex vivo or in vitro cell proliferation can be determined by multiple technologies. The earliest approach is to label cells with [^3^H]-thymidine and quantify proliferation by a gamma counter, which corresponds to DNA synthesis, and has been used to monitor ex vivo immune responses to cancer vaccines. Bromodeoxyuridine (BrdU) incorporation assays use absorbance as the readout and avoid radiolabeled dyes. Other cell proliferation assays use dyes that measure the metabolic activity of cells (eg, MTT (using 3-(4,5-dimethylthiazol-2-yl)-2,5-diphenyltetrazolium bromide), WST-1 (using water-soluble tetrazolium salts)) by permeabilizing the cells and reacting with enzymes or other metabolic factors.

- Assays for monitoring cellular immune responses to active immunotherapy of cancer.^413^
- Immunotherapy of metastatic malignant melanoma by a vaccine consisting of autologous interleukin-2-transfected cancer cells: outcome of a Phase I study.^414^
- Generation of immunity to the HER-2/neu oncogenic protein in patients with breast and ovarian cancer using a peptide-based vaccine.^415^

Fluorescent dyes can be used to monitor cell populations with different rates of division and proliferation. Carboxyfluorescein succinimidyl ester (CFSE) has been commonly used to assess lymphocyte division and proliferation by flow cytometry. CFSE can covalently label intracellular molecules, and CFSE concentration is proportionally diluted in cell progeny according to the number of subsequent cell divisions. DELFIA (dissociation-enhanced lanthanide fluorescence immunoassay) is based on the measurement of time-resolved fluorescence intensity. These fluorescence-based dyes are compatible with multiparametric staining with other fluorochromes.

- The use of carboxyfluorescein diacetate succinimidyl ester (CFSE) to monitor lymphocyte proliferation.^416^
- Role of STAT3 in CD4^+^ CD25^+^ FOXP3^+^ regulatory lymphocyte generation: implications in graft-versus-host disease and antitumor immunity.^417^

The efficiency of in vitro expansion of T cells engineered to express exogenous antigen-specific CAR or TCR measured by proliferation assays can represent a relevant parameter and a surrogate marker of possible in vivo persistence.

- Adoptive cell transfer therapy following non-myeloablative but lymphodepleting chemotherapy for the treatment of patients with refractory metastatic melanoma.^418^
- CAR T cell immunotherapy for human cancer.^419^
- CARs on track in the clinic.^420^

Cytotoxicity assays measure cell death induced by cytotoxic stimuli, environmental changes, or cell-mediated killing. They are based on cell membrane integrity, using vital dyes that allow the exclusion of viable cells, or assessing the release of markers from dying cells (eg, CFSE). In addition, metabolic activity measurements, for example, MTT, lactate dehydrogenase (LDH), or ATP assays, are also used to measure cell viability. A reliable cell-mediated cytotoxicity assay based on radiolabeled ^51^Cr release has been substituted by the development of the assays mentioned above. DELFIA also represents a reliable and simple technology to asses either antibody-dependent cell-mediated cytotoxicity (ADCC) or cell-mediated cytotoxic assays. These assays have been applied to the functional characterization of antitumor and antigen-specific T and NK cell responses, highlighting relevant therapeutic implications.

- Optimization of cytotoxic assay by target cell retention of the fluorescent dye carboxyfluorescein diacetate (CFDA) and comparison with conventional 51CR release assay.^421^
- WT1 peptide-specific T cells generated from peripheral blood of healthy donors: possible implications for adoptive immunotherapy after allogeneic stem cell transplantation.^422^
- Preparation of cytokine-activated NK cells for use in adoptive cell therapy in cancer patients: protocol optimization and therapeutic potential.^423^

##### Conclusions

Cell proliferation and cytotoxic assays can monitor cell-mediated immunity in patients with cancer. Critical considerations include selection of target cells and baseline controls for the assays, and the limitations in applying these techniques to high-throughput screening studies. Nevertheless, in specific cases, for example, characterization of cell-mediated antitumor responses, the integration of cytokine release determination with proliferation and cytotoxic assays should be considered.

#### Assessment of ex vivo antigen-specific immune responses

TAAs are recognized by T cells in the form of MHC/peptide complexes. The discovery and molecular characterization of TAAs allowed the identification and categorization of three main types of antigens.

1. Differentiation antigens that are specific to the cellular lineage. These antigens are overexpressed by tumor cells but also shared with normal tissue (eg, carcinoembryonic antigen (CEA), epithelial cell adhesion molecule (Ep-CAM), mucin-1 (MUC-1), melanoma antigen recognized by T cells-1 (MART-1/Melan-A), glycoprotein 100 (gp100), and tyrosinase (Tyr)).
2. Cancer-Testis (CT) antigens (eg, MAGE, GAGE, LAGE, and NY-ESO-1) are expressed in tumors with different histological origins and their expression in normal tissues is limited to testicular germ cells and placenta.
3. Mutated antigens or neoantigens derived from non-synonymous somatic mutations. These TAAs are not expressed by normal cells and display superior immunogenic potency as compared with differentiation/self or CT antigens. Details of these TAAs have been provided above.

Single or multiple peptides derived from the TAAs mentioned above have been administered in the context of phase I/II clinical studies for vaccination of patients with different types of tumors. However, cancer vaccines have not been associated with durable clinical responses in patients with cancer, possibly as a result of low immunogenic potency and the pre-existence in patients’ body of tolerogenic/anergic T cells specific for these TAAs. Nevertheless, antigen-specific T cell responses could be detected in the circulation of up to 50% of patients with cancer undergoing vaccination. Of note, antigen-specific T cell responses in the circulation of patients with cancer could be detected ex vivo with, in some cases, association with their clinical outcome.

- Progress in the development of immunotherapy for the treatment of patients with cancer^424^
- Cancer/testis antigens, gametogenesis and cancer.^425^
- Therapeutic vaccines for cancer: an overview of clinical trials.^426^

The monitoring of circulating antigen-specific T cell responses in patients with cancer can be performed by ex vivo coculture of peripheral blood lymphocytes with either peptides containing TAA-derived epitopes or HLA-matched antigen-presenting cells loaded with peptides. Cytokine release assays, such as ELISA, ELISPOT, or intracellular staining by flow cytometry, have been used as readouts of antigen-specific reactivity by T cells. These methods have been described above. The majority of studies assessed the effector functions of antigen-specific T cells by measuring the release of IFN-γ, but in some cases also analyzed the multifunctional properties of T cells by assessing the release of multiple cytokines. Multimer staining in combination with flow cytometry has been used to determine the antigen-specific reactivity of T cells.

- Flow cytometric determination of intracellular or secreted IFNgamma for the quantification of antigen reactive T cells.^427^
- Immunologic monitoring of cancer vaccine trials using the ELISPOT assay.^428^
- MHC class II tetramer analyses in AE37-vaccinated prostate cancer patients reveal vaccine-specific polyfunctional and long-lasting CD4^+^ T-cells.^429^
- Anti-CTLA-4 antibody therapy: immune monitoring during clinical development of a novel immunotherapy.^430^

Cancer vaccine studies based on the usage of neoantigens as a source of immunization also showed both the expansion of pre-existing T cell responses and the induction of new T cells reactive against this type of antigen in patients with objective clinical responses.

The ex vivo assessment of antigen-specific reactivity, including neoantigens, could also be successfully performed for TILs, and represented the rationale for ACT protocols for patients with solid tumors (eg, melanoma, CRC, and breast cancer). The efficient recognition by TILs of mutated antigens expressed by autologous tumor cells has been associated with tumor regressions following ACT.

- Somatically mutated tumor antigens in the quest for a more efficacious patient-oriented immunotherapy of cancer.^431^
- Exploiting the curative potential of adoptive T-cell therapy for cancer.^432^
- Tumor exome analysis reveals neoantigen-specific T-cell reactivity in an ipilimumab-responsive melanoma.^120^

##### Conclusions

Ex vivo TAA-specific T cells can be detected in patients with cancer, either as naturally occurring immune responses or as a result of therapeutic interventions through cancer vaccines or immune checkpoint blockade. In some cases, the detection of antigen-specific T cells in the circulation or at the tumor site could be associated with patients’ clinical outcome, and might represent a biomarker for clinical responses to immunotherapy. However, this methodology requires the knowledge of a patient’s HLA type, the availability of sufficient TAA-specific TILs at the tumor site, and the identification and immunogenic validation of HLA-restricted TAA-derived epitopes, and cannot be performed in a high-throughput manner. Development of methods that allow large-scale investigations and standardization is warranted for high-throughput ex vivo assessment of antigen-specific T cell responses.

### Immune contexture biomarker discovery

#### Multiplex immunofluorescent staining

Tissue IHC, a century-old technology, has undergone a major technological revolution in recent years. With the advancement of three major components, (1) biomarker staining methods, (2) whole slide imaging (WSI) techniques, and (3) image analysis software, multiplexing technologies are slowly replacing conventional single-plex IHC assays. Tissue multiplexing technologies play a vital role in understanding the complex TME in cancer immunotherapy. Staining multiple protein biomarkers on a single tissue section facilitates an understanding of complex cell–cell interactions, cell migration and infiltration of immune cells, and cellular distance and density.

#### Staining methods

Three different staining methods are gaining popularity: (1) chromogenic multiplexing, (2) fluorescent multiplexing, and (3) mass spectrometry.

##### Chromogenic multiplexing

Duplex IHC assay: The clinical significance of locating two immune populations (CD3^+^ and CD8^+^) in and around a tumor initiated the concept of Immunoscore, the first validated immune-based assay from FFPE tissue for cancer classification. This standardized assay from HalioDx stains two sequential sections with CD3^+^ and CD8^+^, respectively, scans the slides, and digitally coregisters the markers.

- Biomarkers immune monitoring technology primer: Immunoscore Colon.^21^

The Halioseek assay from HalioDx identifies PD-L1 expression and CD8^+^ populations in the TME from a single slide to help define treatment options for patients with NSCLC. Halioseek is currently a CE-IVD assay (in vitro diagnostic assay certified in the European Economic Area).

In a research setting, a chromogenic multiplex method evaluating 12 biomarkers simultaneously on single FFPE sections of 38 SCCHN cases was used for comprehensive immune phenotyping:

- Quantitative multiplex immunohistochemistry reveals myeloid inflamed tumor-immune complexity associated with poor prognosis.^433^

The availability of new chromogenic dyes of vivid colors is opening up new possibilities for the chromogenic multiplexing field. New series of chromogenic detection kits from Roche (DISCOVERY Teal, DISCOVERY Purple, DISCOVERY Yellow) and Biocare Medical (Viva Green, Bajoran Purple, Ferangi Blue) are entering multiplexing research.

- Covalently deposited dyes: a new chromogen paradigm that facilitates analysis of multiple biomarkers in situ.^434^
- Chromogenic multiplex immunohistochemistry reveals modulation of the immune microenvironment associated with survival in elderly patients with lung adenocarcinoma.^435^

##### Conclusions

Duplex chromogenic assays involving a simple workflow are rapidly entering the clinic to support cancer immunotherapy. However, to develop higher order chromogenic multiplexing assays with a panel of coexpressing biomarkers, advancements in chromogenic dye chemistry, automated stainers, whole slide scanners, and sophisticated image analysis software are necessary and are currently being developed.

#### Fluorescent multiplexing

Multiplex immunofluorescent staining technology allows multiple biomarkers tagged with distinct fluorescent dyes to be interrogated separately or in combination, and has exhibited exponential growth in recent years. Although fluorescent multiplex assays are predominantly research use only or lab-derived test, future clinical adoption is anticipated. The technology requires investment in fluorescent microscopes and scanners, and relies heavily on image analysis software for downstream analysis and effective outcomes.

This section highlights three different fluorescent staining methodologies:

##### Simultaneous multiplexing

The simultaneous multiplexing staining method involves the application of a cocktail of primary and secondary antibodies to the tissue, thereby simplifying the staining process and saving time.

The UltraPlex staining technology from Cell IDx can detect four to six biomarkers in a single tissue section using a simultaneous multiplexing method. The staining assay uses the standard two-step staining process where cocktails of primary and secondary antibodies are used. Each primary antibody is conjugated to a unique modified hapten and each hapten-specific secondary antibody is labeled with a distinct fluorescent dye.

- Hapten-anti-hapten technique for two-color IHC detection of phosphorylated EGFR and H2AX using primary antibodies raised in the same host species.^436^

The InSituPlex staining technology from Ultivue involves the repeated application of antibody cocktails (up to four antibodies per round of staining) labeled with unique DNA barcodes to a tissue section, which, following an amplification step, are detected by hybridization with complementary DNA barcodes tagged with distinct fluorophores.

- DNA barcoded labeling probes for highly multiplex Exchange-PAINT imaging.^437^

The CODEX (CO-Detection by indEXing) staining technology from AKOYA Biotechnology enables higher orders of multiplexing (39-plex) on a single tissue section. Here, the primary antibodies are conjugated to proprietary barcodes, each with its unique oligonucleotide sequence. The dye-labeled reporter targets the barcode with high specificity. Although the primary antibody staining is a single step, signals are detected by sequential scanning and imaging of three fluorescent reporters in each round.

- Deep profiling of mouse splenic architecture with CODEX multiplexed imaging.^438^

Morphology-driven higher order multiplexing of the tumor inflammation signature that simultaneously measures DNA, RNA, and protein using GeoMax DSP technology is available from NanoString.

- New tools for pathology: a user’s review of a highly multiplexed method for in situ analysis of protein and RNA expression in tissue.^439^

##### Sequential multiplexing

The sequential multiplex-staining method involves multiple (5 to 8) biomarker detection on a single tissue section. The most commonly used method consists of staining with unmodified primary antibody and horseradish peroxidase-conjugated secondary antibody followed by enzyme-mediated deposition of tyramide-fluorophores on the epitope of interest. A heat deactivation step is applied between each staining round. Recent adoption of these staining protocols to automated staining platforms (DISCOVERY ULTRA from Roche, BOND RX from Leica Biosystems) has reduced the staining time significantly. Two commonly used sequential multiplexing staining methods are described here:

- Fully automated 5-plex fluorescent immunohistochemistry with tyramide signal amplification and same species antibodies.^440^
- An automated staining protocol for seven-color immunofluorescence of human tissue sections for diagnostic and prognostic use.^441^

##### Cyclic multiplexing technology

The cyclic multiplexing technology MultiOmyx from NeoGenomics (originally developed by GE Global Research) is capable of higher order multiplexing of up to 60 biomarkers on a single tissue section. Each staining/detection round involves tissue incubation with a cocktail of two to four primary antibodies directly conjugated to unique fluorescent dyes, followed by tissue imaging, chemical deactivation of the fluorescent dyes, and rescanning to capture the background image. This technology requires dedicated scanners and image analysis platforms.

- Highly multiplexed single-cell analysis of formalin-fixed, paraffin-embedded cancer tissue.^442^

#### Mass spectrometry for tissue multiplexing

Mass spectrometry is used for identifying multiple biomarkers from a single tissue section. Typically, the tissue section is incubated with a cocktail of antibodies tagged with unique metal tags, each targeting a different protein of interest. Such methodologies are supported by dedicated imaging systems and image analysis software. Two technologies available in the market are highlighted here.

##### Imaging mass cytometry (IMC)

Fluidigm has combined mass cytometry or CyTOF with the Hyperion Imaging System to perform multiplexing on tissue sections. IMC on the Hyperion Imaging System enables simultaneous analysis of 4–37 protein markers from a single tissue scan. The process, however, leads to vaporization of the biological material during the scan.

- histoCAT: analysis of cell phenotypes and interactions in multiplex image cytometry data.^443^

##### Multiplexed ion beam imaging (MIBI) technology

The MIBI technology from IONpath uses a cocktail of antibodies, each tagged to different lanthanides for tissue staining. The ion beam imaging scope (MIBIscope can identify up to 40+ markers simultaneously from the tissue. The sample undergoes raster scanning with an ion beam that allows multiple rescannings of the tissue at different resolutions.

- Structured tumor-immune microenvironment in triple negative breast cancer revealed by multiplexed ion beam imaging.^444^

##### Conclusions

Multiple multiplexing methods are available to identify up to hundreds of biomarkers from a single tissue section. However, most of these technologies are extensively used in biomarker exploratory studies for cancer immunotherapy. Over time, the immunotherapy community may adopt a few key technologies for clinical practice.

#### Whole slide imaging and image analysis

##### Whole slide scanners

Although various multiplexing staining techniques are currently available, the staining outcome is heavily dependent on scanning platforms and downstream analysis software. In the field of immunotherapy, the general consensus is leaning toward WSI, as it is more informative than a selected number of regions of interest (ROIs) from a stained tissue section. However, WSI is limited by throughput and remains one of the key bottlenecks in the turnaround time of the workflow.

Some tissue multiplexing technologies provide complementary scanners along with an image analysis platform such as CODEX from AKOYA Biosciences, MultiOmyx from NeoGenomics, and mass spectrometry multiplexing from Fluidigm and IONpath.

Multi-filter-based fluorescent whole slide scanners currently available in the market include the Zeiss Axio Scan Z1, NanoZoomer series from Hamamatsu, Aperio VERSA from Leica Biosystems, Olympus V120, and so on. The Vectra and Polaris scanner series from Phenoptics (AKOYA Biosciences) have additional multispectral imaging capabilities. These research instruments are capable of performing both bright field and fluorescent WSI scans.

Philips IntelliSite Pathology Solution is the first WSI system approved (in 2017) by the FDA for primary diagnosis in surgical pathology. Some of the key players in the space are Roche, Leica Biosystems, 3DHistech, and Hamamatsu. Clinical implications and the future of WSI are discussed in the following paper.

- US Food and Drug administrative approval of whole slide scan for primary diagnosis: a key milestone is reached or new questions are raised.^445^

##### Image analysis platforms

Various image analysis software systems are tailored for analyzing multiplex slides. Indica Lab’s HALO contains a multiplex IHC module for chromogenic multiplex and a Highplex FL module for fluorescent multiplex image analysis. Visiopharm offers the Phenomap multiplexing image analysis software, while Phenoptics’ inForm image analysis software is another commonly used tool. Some additional analysis platforms are AQUAnalysis from HistoRx, MultiOmyx from NeoGenomics, HistoQuant from 3DHistech, and Tissue Studio from Definiens.

With the advent of artificial intelligence (AI) in the digital pathology space, rapid developments are anticipated. The challenges and opportunities of the field have been described in the following review articles.

- Artificial intelligence and digital pathology: Challenges and opportunities.^446^
- Artificial intelligence in digital pathology—new tools for diagnosis and precision oncology.^447^

##### Conclusions

The availability of new tools and technologies is opening up new options for WSI and image analysis. However, major improvements are required in workflow scalability, high-throughput scanners, ease of use protocols, scan turnaround time, high-quality image capture, and rapid image analysis algorithms. Computer vision and AI may transform the digital pathology landscape in the next few years.

#### Application of tissue multiplexing technology

Different groups have successfully applied the complex multiplexing workflow to answer key questions in the field of cancer immunotherapy. The following are some of the recent publications.

- Systems pathology by multiplexed immunohistochemistry and whole-slide digital image analysis.^448^
- Validation of multiplex immunofluorescence panels using multispectral microscopy for immune-profiling of formalin-fixed and paraffin-embedded human tumor tissues.^449^
- Multispectral fluorescence imaging allows for distinctive topographic assessment and subclassification of tumor-infiltrating and surrounding immune cells.^450^
- Multiplex immunohistochemistry for molecular and immune profiling in lung cancer-just about ready for prime time?^451^

A comprehensive review of complex tissue multiplexing technology is provided here.

- State-of-the-art of profiling immune contexture in the era of multiplexed staining and digital analysis to study paraffin tumor tissues.^452^

##### Conclusions

Tissue multiplexing technology holds promise for clinical adoption. Chromogenic duplex assays, a whole slide scanner, and digital pathology algorithms have obtained clinical approval in recent years. Maturation of this field will provide enormous benefits for the field of cancer immunotherapy.

### Software and tools for data analysis

#### Gene expression analysis tools

Data analyses of gene expression profiling can be categorized depending on the study objectives, that is, class discovery, class comparison, class prediction, and survival analysis.

- Design and analysis of DNA microarray investigations.^453^

Class discovery is an unsupervised method with the goal of discovering clusters among specimens or among genes. Hierarchical clustering algorithms, principal component analysis, a self-organizing map, and non-negative matrix factorization are popular methods to find clusters of samples or genes.

- Cluster analysis and display of genome-wide expression patterns.^454^
- Principal components analysis to summarize microarray experiments: application to sporulation time series.^455^
- Analysis of gene expression data using self-organizing maps.^456^
- Knowledge-based gene expression classification via matrix factorization.^457^

Class comparison aims to identify genes differentially expressed between two or more different tissue types or experimental conditions. For microarray gene expression data, a Student’s t-test is generally used to identify significantly differentially expressed genes between two phenotype classes, assuming the log-transformed ratio or intensity data are approximately normally distributed. A multiple testing problem occurs during class comparison due to the simultaneous testing of all genes. The false discovery rate method is commonly used to control for false discoveries in a set of identified genes.

- Microarrays, empirical Bayes methods, and false discovery rates.^458^
- Significance analysis of microarrays applied to transcriptional responses to ionizing radiation.^459^
- Controlling the false discovery rate: a practical and powerful approach to multiple testing.^460^

Class prediction is used to predict the phenotype of new samples from their gene expression. Popular prediction methods include diagonal linear discriminant analysis, K-nearest neighbors, support vector machine, and random forest. Lasso and least angle regression (LARS) can be used to build prediction models with continuous outcomes. Cross validation needs to be properly performed to avoid overfitting, and the final model always needs to be evaluated on independent data sets.

- Regularized linear discriminant analysis and its application in microarrays.^461^
- Diagnosis of multiple cancer types by shrunken centroids of gene expression.^462^
- Gene selection for sample classification based on gene expression data: study of sensitivity to choice of parameters of the GA/KNN method.^463^
- Knowledge-based analysis of microarray gene expression data by using support vector machines.^464^
- Gene selection and classification of microarray data using random forest.^465^
- The elements of statistical learning: data mining, inference, and prediction (Springer Series in Statistics).^466^
- The lasso method for variable selection in the Cox model.^467^
- A paradigm for class prediction using gene expression profiles.^468^

Gene expression-based survival analyses address two major areas: (1) identifying signatures that are associated with survival, and (2) building a prognostic and/or predictive model based on survival data. One method uses a Cox proportional hazards model to relate survival time to k ‘supergene’ expression groups. The ‘supergene’ expression levels are the first k principal component, that is, linear combinations of expression levels of the subset of genes that are univariately correlated with survival. Another method uses a penalized Cox regression model to find genes related to survival, which can be done using the R package glmnet.

- Diagnostic and prognostic prediction using gene expression profiles in high-dimensional microarray data.^469^
- ‘Gene shaving’ as a method for identifying distinct sets of genes with similar expression patterns.^470^
- Regularization paths for Cox’s proportional hazards model via coordinate descent.^471^

The R statistical programming language together with the Bioconductor repository provides over 1000 packages for the analysis of high-throughput genomic data including gene expression. For users with limited or no programming skills, a more suitable choice may be GEO2R, which can provide simple data analysis on a curated National Center for Biotechnology Information (NCBI) GEO data set. Such users can also use a general-purpose software that can handle more sophisticated data analyses. For example, BRB-ArrayTools is a Windows desktop application with a graphical interface designed for use by researchers who want to use state-of-the-art statistical methods for gene expression analysis.

- Bioinformatics and computational biology solutions using R and Bioconductor.^472^
- GEOquery: a bridge between the Gene Expression Omnibus (GEO) and BioConductor.^473^
- Analysis of gene expression data using BRB-ArrayTools.^474^

#### WES and RNA-seq data analysis tools

The first step in the workflow of WES/RNA-seq data is mapping. Raw sequence data are commonly saved in the FASTQ format, which needs to be mapped to a reference genome by using an alignment algorithm. Popular mapping/alignment tools include Burrows-Wheeler Aligner (BWA)-Maximal Exact Match (MEM) and Subread for DNA-seq data, and TopHat2 and spliced transcripts alignment to a reference (STAR) for RNA-seq data.

- TopHat2: accurate alignment of transcriptomes in the presence of insertions, deletions and gene fusions.^475^
- STAR: ultrafast universal RNA-seq aligner.^476^
- The Subread aligner: fast, accurate and scalable read mapping by seed-and-vote.^477^
- Aligning sequence reads, clone sequences and assembly contigs with BWA-MEM.^478^

Next, variant calling can be conducted on both aligned RNA-seq and DNA-seq data using Samtools or Genome Analysis Toolkit best practices pipeline tools. For WES data with tumor/normal pairs, somatic mutations are called using tools such as MuTect (for SNPs) and Mutect2 (for indels). The variant call format files containing variant information are finally annotated using tools such as ANNOVAR, SnpEff, and variant effect predictor (VEP).

- From FastQ data to high confidence variant calls: the Genome Analysis Toolkit best practices pipeline.^479^
- The sequence alignment/map format and SAMtools.^480^
- Sensitive detection of somatic point mutations in impure and heterogeneous cancer samples.^481^
- ANNOVAR: functional annotation of genetic variants from high-throughput sequencing data.^482^
- A program for annotating and predicting the effects of single nucleotide polymorphisms, SnpEff: SNPs in the genome of Drosophila melanogaster strain w1118; iso-2; iso-3.^483^
- The Ensembl variant effect predictor.^484^

Further levels of analyses based on WES data include using tools such as Sequenza to generate allele-specific copy numbers from the mapped reads and PyClone to generate the tumor clonal distribution. To get an estimate of tumor purity and ploidy, fraction and allele-specific copy number estimates from tumor sequencing (FACETS) can be applied on mapped reads. In addition, PolySolver can be used to predict patient HLA types, and the MSI status can be detected using a method called microsatellite instability by NGS (mSINGS). Finally, multiple pipelines have been developed to predict neoantigens based on annotated variants in the translated protein sequence.

- Sequenza: allele-specific copy number and mutation profiles from tumor sequencing data.^485^
- PyClone: statistical inference of clonal population structure in cancer.^486^
- FACETS: allele-specific copy number and clonal heterogeneity analysis tool for high-throughput DNA sequencing.^487^
- Comprehensive analysis of cancer-associated somatic mutations in class I HLA genes.^488^
- Microsatellite instability detection by next generation sequencing.^489^
- Applications of immunogenomics to cancer.^490^

To generate quantitative measurements from the aligned RNA-sequence data, the high-throughput sequencing (HTSeq) method and featureCounts software can be applied to generate count data. Similar tools include RNA-seq by expectation maximization (RSEM) and Salmon. Due to the nature of RNA-seq data, differential expression analysis for sequence count data (DESeq), DESeq2, Cuffdiff, and EdgeR use a negative binomial distribution with generalized linear models to determine the significance of genes that are differentially expressed between classes. The mapped reads from RNA-seq data can be further explored to study the immune repertoire of the TCR and B cell receptor using tools such as TCR repertoire utilities for solid tissue/tumor (TRUST), and to analyze immune cell composition using tools such as cell type identification by estimating relative subsets of RNA transcripts (CIBERSORT) and the tumor immune estimation resource (TIMER).

- HTSeq-a Python framework to work with high-throughput sequencing data.^491^
- featureCounts: an efficient general-purpose program for assigning sequence reads to genomic features.^492^
- RSEM: accurate transcript quantification from RNA-seq data with or without a reference genome.^493^
- Salmon provides fast and bias-aware quantification of transcript expression.^494^
- Differential expression analysis for sequence count data.^495^
- Moderated estimation of fold change and dispersion for RNA-seq data with DESeq2.^496^
- edgeR: a Bioconductor package for differential expression analysis of digital gene expression data. Bioinformatics.^497^
- Differential gene and transcript expression analysis of RNA-seq experiments with TopHat and Cufflinks.^498^
- Antigen receptor repertoire profiling from RNA-seq data.^499^
- Profiling tumor infiltrating immune cells with CIBERSORT.^500^
- Comprehensive analyses of tumor immunity: implications for cancer immunotherapy.^501^

For scRNA-seq data, scalability and technical noise are two main bioinformatics challenges. Certain methods that were developed for bulk cell RNA-seq cannot be applied directly and require adaptation. Many methods and tools have been developed specifically for scRNA-seq data. For example, Scater, SCnorm, Seurat, and SCANPY are R or Python packages developed to facilitate rigorous preprocessing, quality control, normalization, and visualization of scRNA-seq data. Depending on the experimental design, protocol, and platform, SCONE uses a data-driven approach to allow the user to select an appropriate normalization strategy for scRNA-seq data. Falco is a cloud-based scRNA-seq processing framework which provides a scalable and efficient computational solution. For data visualization, many tools, such as t-distributed stochastic neighbor embedding (t-SNE), uniform manifold approximation and projection (UMAP), and single-cell interpretation via multi-kernel learning (SIMLR) can be used as a dimensionality reduction step for visualizing scRNA-seq data in two dimensions. A typical scRNA-seq analysis by Luecken and Theis^502^ serves as a strong example of best practices for this technique.

- Computational and analytical challenges in single-cell transcriptomics.^291^
- Scater: pre-processing, quality control, normalization and visualization of single-cell RNA-seq data in R.^503^
- SCnorm: robust normalization of single-cell RNA-seq data.^504^
- Integrating single-cell transcriptomic data across different conditions, technologies, and species.^505^
- SCANPY: large-scale single-cell gene expression data analysis.^506^
- Performance assessment and selection of normalization procedures for single-cell RNA-seq.^507^
- Falco: a quick and flexible single-cell RNA-seq processing framework on the cloud.^508^
- Dimensionality reduction for visualizing single-cell data using UMAP.^509^
- Visualization and analysis of single-cell RNA-seq data by kernel-based similarity learning.^510^
- Current best practices in single-cell RNA-seq analysis: a tutorial.^502^

#### Multiparameter cytometric data analysis

One of the main goals of multiparameter cytometry data analysis is to identify the proportion of immune cell subpopulations (eg, memory CD8^+^ T cells, Th1 cells, and so on) and their properties (eg, expression of activation markers or cytokines, antigen-specificity of T cells, and so on).

Analysis of flow cytometry data starts from data preprocessing (compensation, batch effect assessment and removal, curation of data sets, and exclusion of dead cells and doublets) followed by placing the ‘gates’ to identify cell subsets and obtain property information of those subsets. Postprocessing entails comparison of groups and aims to find significantly altered subpopulations of cells with appropriate statistical methods.

- Guidelines for the use of flow cytometry and cell sorting in immunological studies.^511^

Traditionally, flow cytometric data are manually analyzed using a dedicated software with a graphical user interface, for example:

- https://www.flowjo.com/512
- https://www.denovosoftware.com/513
- https://www.aceabio.com/products/novoexpress-software/514
- https://www.vsh.com/products/winlist/index.asp^515^
- https://www.miltenyibiotec.com/US-en/products/macs-flow-cytometry/software/flowlogic-software.html^516^
- https://www.beckman.com/flow-cytometry/software/kaluza^517^

Similar to flow cytometry data analysis, mass cytometry data also require preprocessing (eg, transformation of ion counts, normalization and batch correction, and so on).

- The anatomy of single cell mass cytometry data.^365^

Recent developments in both multiparameter flow and mass cytometry enable the interrogation of up to 40 parameters at the single cell level. This high dimensionality requires sophisticated computational approaches when analyzing data to gain worthwhile insights from information-rich data sets. As the list of available analysis tools continues to grow, it is worthwhile to start from a high-level overview. There are supervised and unsupervised approaches. We will cover unsupervised approaches first, followed by supervised approaches.

The most commonly used unsupervised analyses are nicely summarized here.

- Algorithmic tools for mining high-dimensional cytometry data.^518^
- Computational flow cytometry: helping to make sense of high-dimensional immunology data.^519^
- The end of gating? An introduction to automated analysis of high dimensional cytometry data.^520^

Unsupervised approaches for multiparametric flow or mass cytometry have a significant overlap with those for single-cell NGS data sets. In general, these can be classified as (1) data visualization, (2) cell type identification, (3) differential analysis, and (4) network and multiomics data integration.

- A beginner’s guide to analyzing and visualizing mass cytometry data.^521^
- Computational approaches for high-throughput single-cell data analysis.^522^
- Advancing systems immunology through data-driven statistical analysis.^523^
- Mass cytometry: a powerful tool for dissecting the immune landscape.^524^

While most analysis tools are written in open source programing languages such as R, Python, or others, fee-based platform services are available for multiparametric cytometry data.

- https://www.cytobank.org/525
- https://www.astrolabediagnostics.com/526

Benchmark assessment of algorithms is a helpful resource when deciding what algorithms to use, although it is recommended to use multiple algorithms to ensure the validity of analysis results.

- Critical assessment of automated flow cytometry data analysis techniques.^527^
- Comparison of clustering methods for high-dimensional single-cell flow and mass cytometry data.^528^

A supervised approach is also useful if population and biomarker candidates are well defined with the traditional cascaded gating strategy. In this case, automated gating reduces both interanalysts’ variabilities and time spent for manual gating.

- Flow cytometry bioinformatics.^529^
- Standardizing flow cytometry immunophenotyping analysis from the human immunophenotyping consortium.^530^
- A standardized immune phenotyping and automated data analysis platform for multicenter biomarker studies.^531^
- Implementation and validation of an automated flow cytometry analysis pipeline for human immune profiling.^532^

#### Software and tools for function and pathway analysis

Functional enrichment and pathway analysis are essential tasks for the interpretation of gene lists derived from large-scale genetic, transcriptomic and proteomic studies. Two of the most popular resources for understanding high-level functions and utilities of the biological system are the Kyoto Encyclopedia of Genes and Genomes (KEGG) and BioCarta. A comprehensive list of available tools is provided at the Gene Ontology website. ImmuneSigDB is a comprehensive compendium of 5000 gene sets pertaining to immune biology, which may provide the systems immunologist with a useful resource for analysis of gene expression in the immune system.

- http://www.geneontology.org^533^
- KEGG: Kyoto Encyclopedia of Genes and Genomes.^534^
- The Gene Ontology (GO) database and informatics resource.^535^
- Compendium of immune signatures identifies conserved and species-specific biology in response to inflammation.^536^

Genomic, proteomic, and metabolomic data typically result in lists of interesting genes or proteins. Translating these gene sets into an understanding of the underlying biological mechanism is a fundamental need in biological research. A popular resource that can help unravel the mechanism behind a specific set of mutations is the Database for Annotation, Visualization, and Integrated Discovery (DAVID). Pathway Commons is another tool used by computational biologists to download custom subsets of pathway data for analysis, or to incorporate powerful biological pathway and network information retrieval and query functionality into websites and software. One widely used commercial product is Ingenuity Pathway Analysis, which allows the user to access many different algorithms to identify the most significant pathways and discover potential novel regulatory networks and causal relationships associated with experimental data.

- DAVID: https://david.ncifcrf.gov^537^
- Pathway Commons: http://www.pathwaycommons.org/pc2^538^
- DAVID bioinformatics resources: expanded annotation database and novel algorithms to better extract biology from large gene lists.^539^
- Pathway Commons, a web resource for biological pathway data.^540^
- Causal analysis approaches in Ingenuity Pathway Analysis.^541^

Gene set enrichment analysis (GSEA) is a computational method that determines whether a specific set of genes shows statistically significant, concordant differences between two biological states. The gene-level statistics for all genes in a pathway are aggregated into a single pathway-level statistic. Finally, statistically significant pathways can be identified. The tools for this kind of analysis are distributed as either stand-alone desktop applications or as packages for R (eg, R-GSEA). Gene set analysis (GSA) is an R function that differs from GSEA in its use of the ‘maxmean’ statistic, which is often more powerful than the modified Kolmogorov-Smirnov statistic used in GSEA. GSA can also handle more than two groups, such as multiple classes, survival times, and quantitative outcomes. Both GSEA and GSA approaches can help identify the most promising pathways or gene sets to be used as predictive or prognostic biomarkers in immunotherapy.

- Ten years of pathway analysis: current approaches and outstanding challenges.^542^
- Gene set enrichment analysis: a knowledge-based approach for interpreting genome-wide expression profiles.^543^
- Exploring gene expression data with class scores.^544^
- On testing the significance of sets of genes.^545^

#### Conclusions

The advancement of high-throughput technologies has provided an unprecedented opportunity to conduct comprehensive analyses of genes, transcripts, proteins, and other significant biological molecules for biomarker identification. However, it has also complicated the process of finding meaningful markers from these complex data sets. It is time-consuming and challenging to validate the accuracy of each step involved in the analysis workflow. In order to obtain reproducible results, it is imperative to harmonize not only sample preparation and assay execution protocols but also data analysis procedures.

Many software packages and pipelines have been developed that have allowed us to effectively process WES/RNA-seq data and multiparameter cytometry data, as well as to perform gene set/pathway analyses. However, depending on the methods used in each data processing and analysis step, substantially different results and conclusions may be developed from the same data set. These results should be assessed and validated carefully with different methods and experiments.

### In vivo imaging (non-invasive and whole body)

Strict anatomical imaging criteria may be insufficient to cover the spectrum of response to immunotherapy. Updated immune-related response criteria are required that incorporate imaging patterns observed with immunotherapy.

- Guidelines for the evaluation of immune therapy activity in solid tumors: immune-related response criteria.^546^
- Personalized tumor response assessment in the era of molecular medicine: cancer-specific and therapy-specific response criteria to complement pitfalls of RECIST.^547^

Tumor metabolic processes precede structural changes in anatomical imaging and as such may provide sensitive indicators of early response to therapy. It remains a challenge, however, to distinguish between neoplasms and infectious or inflammatory processes. This is particularly problematic in the midst of irAEs.

- Ipilimumab-induced immune-mediated adverse events: possible pitfalls in ^18^F-FDG-PET/CT interpretation.^548^
- Bacillus Calmette-Guerin injections for melanoma immunotherapy: potential for a false-positive PET/CT.^549^

There are relatively few clinical trials involving immunotherapy that include molecular imaging, but those that do target immune cells or functional markers.

#### Imaging cytotoxic lymphocytes

- Synthesis of 2’-deoxy-2’-[^18^F]fluor-9-beta-D-arabinofuranosylguanine: a novel agent for imaging T cell activation with PET.^550^
- Molecular imaging of lymphoid organs and immune activation by positron emission tomography with a new ^18^F-labeled 2’-deoxycytidine analog.^551^
- Noninvasive detection of therapeutic cytolytic T cells with ^18^F-FHBG PET in a patient with glioma.^552^

#### Imaging immunosuppressive factors and cells

- TGF-β antibody uptake in recurrent high-grade glioma imaged with ^89^Zr-fresolimumab PET.^553^
- Clinical applications of iron oxide nanoparticles for magnetic resonance imaging of brain tumors.^554^
- Antibody positron emission tomography imaging in anticancer drug development.^555^

Preclinical studies continue to explore molecular imaging probes that enable visualization of immune responses within tumors. Immuno-PET, the use of antibodies or antibody fragments to target PET radionuclides, introduces increased specificity to imaging, with new probes appearing with increasing frequency.

- An effective immuno-PET imaging method to monitor CD8-dependent responses to immunotherapy.^556^

Antibody engineering optimizes in vivo pharmacokinetics and provides improved blood clearance for imaging at early timepoints with high tissue specificity.

- Engineered antibody fragments for immuno-PET imaging of endogenous CD8^+^ T cells in vivo.^557^
- Targeting T and B lymphocyte with radiolabeled antibodies for diagnostic and therapeutic applications.^558^
- Noninvasive imaging of immune responses.^559^

Immuno-PET probes targeted to cytokines (eg, IL-1β and IFN-γ) show increased tissue specificity over probes targeted at immune cell types that show significant uptake in secondary lymphoid tissues.

- Immuno-PET of innate immune markers CD11b and IL-1β detect inflammation in murine colitis.^560^
- IFN-γ PET Imaging as a predictive tool for monitoring response to tumor immunotherapy.^561^

Radiolabeling of anti-PD-L1 antibodies and small non-antibody therapeutics allows for the in vivo distinction of PD-L1 positive and negative tumors.

- Noninvasive imaging of tumor PD-L1 expression using radiolabeled anti-PD-L1 antibodies.^562^
- Engineering high affinity PD-1 variants for optimized immunotherapy and immuno-PET imaging.^563^

Given the intensity of work on CAR T cell therapy, significant efforts are underway to generate reporter systems to track the distribution, persistence, and in situ function of transferred T cells.

- [^18^F]FHGB PET/CT imaging of CD34-TK75 transduced donor T cells in relapsed allogeneic stem cell transplant patients: safety and feasibility.^564^
- Quantitative imaging of the T cell antitumor response by positron-emission tomography.^565^
- Imaging TCR-dependent NFAT-mediated T-cell activation with positron emission tomography in vivo.^566^

Finally, a growing mechanistic understanding of T cell metabolism in TME enables in vivo quantification of effector T cell accumulation pretherapy and post-therapy through metabolite detection.

- Lactate chemical exchange saturation transfer (LATEST) imaging in vivo a biomarker for LDH activity.^567^
- Molecular imaging biomarkers for cell-based immunotherapies.^568^

#### Conclusions

Molecular imaging allows non-invasive profiling of whole primary tumors, distal metastases, and involved lymph nodes, potentially aiding patient selection for given therapies and evaluation of response. Molecular imaging is just starting to be used to monitor therapy in immuno-oncology, and preclinical models indicate the potential to monitor specific cellular processes in a longitudinal manner independent of biopsy bias.

### Predictive metabolic biomarkers in tumor immunotherapy

While select metabolic enzymes and their byproducts have been investigated as prognostic biomarkers in various cancers, the use of metabolic biomarkers as predictive guides for cancer immunotherapy is an emerging concept that is currently in its infancy. There is a significant amount of evidence supporting the role of the metabolic enzyme, indoleamine 2,3-dioxygenase (IDO-1), in immune tolerance. However, the ECHO-301/KEYNOTE-252 study recently showed no significant improvement in the clinical outcome of patients with stage IV melanoma with the addition of the IDO-1-selective inhibitor, epacadostat, to pembrolizumab (anti-PD-1 antibody) compared with pembrolizumab alone. In addition to the emerging field of immunometabolism and the realization that metabolic pathways play critical roles in directing immune cell function, the results of this clinical trial support the need for additional studies designed to identify metabolic biomarkers capable of predicting responses to immunotherapeutic combination regimens.

#### Tryptophan-degrading enzymes

The metabolic by-products of tryptophan (Trp) degradation including kynurenine (kyn) have been implicated as playing an important role in the generation of immune tolerance. Given that IDO-1 is upregulated in more inflamed environments due to its regulation by IFN signaling, expression levels often correlate with PD-L1 expression and numbers of infiltrating CD8^+^ T cells. As a result, several studies have found positive associations between IDO-1 expression and response to checkpoint blockade.

- A prospective phase II trial exploring the association between tumor microenvironment biomarkers and clinical activity of ipilimumab in advanced melanoma.^141^
- An immune-active tumor microenvironment favors clinical response to ipilimumab.^569^
- High IDO-1 expression in tumor endothelial cells is associated with response to immunotherapy in metastatic renal cell carcinoma.^570^

These data seemingly contradict additional studies that have also linked IDO-1 expression by certain tumor models with resistance to anti-CTLA-4 antibody immunotherapy. Consistent with this finding, IDO-1 can promote the recruitment of myeloid-derived suppressor cells (MDSCs), making these tumors more susceptible to agents blocking MDSC recruitment.

- Indoleamine 2, 3-dioxygenase is a critical resistance mechanism in antitumor T cell immunotherapy targeting CTLA-4.^571^
- Targeting myeloid-derived suppressor cells with colony stimulating factor-1 receptor blockade can reverse immune resistance to immunotherapy in indoleamine 2,3-dioxgenase-expressing tumors.^572^

The IDO-1 enzyme converts Trp to a series of metabolic by-products including kyn. While this process starves effector T cells of the essential amino acid Trp and suppresses their proliferation, kyn drives regulatory T cell differentiation and promotes immune tolerance. Indeed, the following reference describes serum kyn/Trp ratios and quinolinic acid concentrations based on high-performance liquid chromatography-mass spectrometry (HPLC-MS) and MS analyses as being associated with diminished PFS in patients with NSCLC undergoing nivolumab (anti-PD-1 antibody) immunotherapy.

- Can IDO activity predict primary resistance to anti-PD-1 treatment in NSCLC?.^573^

Notably, these data are also consistent with a recent study demonstrating that adaptive increases in serum kyn/Trp ratio are associated with inferior OS in patients with advanced melanoma and RCC undergoing nivolumab immunotherapy.

- Metabolomic adaptations and correlates of survival to immune checkpoint blockade.^574^

#### Adenosine

Adenosine is an immunosuppressive nucleoside that has been demonstrated to negatively regulate antitumor immunity by directly suppressing effector T cell and NK cell functions. CD73 is an ecto-5’-nucleotidase that controls levels of adenosine in the TME by catalyzing the breakdown of adenosine monophosphate (AMP). Preclinical studies indicate that tumor expression of CD73 is associated with inferior responses to checkpoint inhibitor immunotherapy and increased responsiveness to the inhibition of adenosine signaling. CD73 can also be cleaved from the cell surface and found in the serum. Recent work has shown that the enzymatic activity of soluble CD73 in the serum correlates with response to nivolumab in patients with metastatic melanoma.

- Antimetastatic effects of blocking PD-1 and the adenosine A2A receptor.^575^
- Adenosine receptor 2A blockade increases the efficacy of anti-PD-1 through enhanced antitumor T cell responses.^576^
- Soluble CD73 as biomarker in metastatic melanoma patients treated with nivolumab.^577^

#### Tumor glycolysis and oxidative phosphorylation

The metabolic landscape of the TME has been shown to contribute to immunotherapy resistance in a variety of contexts. The elevated glycolytic capacity of malignant tissues has been associated with T cell glucose starvation and diminished antitumor immune responses. Indeed, circulating levels of LDH, which may serve as a surrogate for glycolytic levels in the TME, also correlate with inferior responses to checkpoint inhibitor therapy.

- Increased tumor glycolysis characterizes immune resistance to adoptive T cell therapy.^578^
- Targeting tumor-associated acidity in cancer immunotherapy.^579^

Other studies have indicated that the process of oxidative phosphorylation can also be associated with resistance to anti-PD-1 blockade in both melanoma cell lines and clinical melanoma specimens.

- Tumor cell oxidative metabolism as a barrier to PD-1 blockade immunotherapy in melanoma.^580^

Oxidative phosphorylation of tumors has been associated with the development of tumor hypoxia, a process that has also correlated with resistance to pembrolizumab immunotherapy in preclinical models and patients with advanced melanoma.

- Genomic and transcriptomic features of response to anti-PD-1 therapy in metastatic melanoma.^166^
- Efficacy of PD-1 blockade is potentiated by metformin-induced reduction of tumor hypoxia.^581^

Fatty acid oxidation (FAO) drives oxidative phosphorylation and in local myeloid cells within the TME this process may contribute to tumor-mediated immune evasion. Consistent with an important role for FAO in the regulation of tumor immunity, the genetic silencing and systemic pharmacological inhibition of this pathway in preclinical models enhance the efficacy of immunotherapy.

- Inhibition of fatty acid oxidation modulates immunosuppressive functions of myeloid-derived suppressor cells and enhances cancer therapies.^582^
- Paracrine Wnt5a-β-catenin signaling triggers a metabolic program that drives dendritic cell tolerization.^583^

#### Conclusions

In light of the emerging data highlighting the critical role of cellular metabolism in the regulation of antitumor immunity, the role of metabolic biomarkers in the development of novel immunotherapy strategies is likely to expand. The development of methods to more readily study in situ metabolic biomarkers in a cell type-specific manner would greatly facilitate the use of metabolic biomarkers in clinical trial development.

**Table 2:** Online resources: tools for the bench and other useful websites

Resource	Description	URL link
CIMAC/CIDC network	The Cancer Immune Monitoring and Analysis Centers (CIMAC) and the Cancer Immunologic Data Commons (CIDC) are NCI-funded academic centers for advanced clinical trial immune monitoring.	https://cimac-network.org/
PACT	The Partnership for Accelerating Cancer Therapies (PACT) is a public–private collaboration that extends the CIMAC/CIDC activities to include additional non-NCI clinical trials.	https://fnih.org/what-we-do/programs/partnership-for-accelerating-cancer-therapies
Links to FDA biomarker approval	The FDA’s Center for Drug Evaluation and Research works with stakeholders to identify and develop new biomarkers, review biomarkers for use in regulatory decision-making, and qualify biomarkers for specific contexts of use.	https://www.fda.gov/drugs/drug-development-tool-qualification-programs/cder-biomarker-qualification-program
Public databases	ImmPort is a data repository and sharing tool built by NIAID for immunology-related assay data of various types.	http://www.immport.org
	The Cancer Genome Atlas is a database of sequences from over 20,000 cancer and matched normal tissues.	https://portal.gdc.cancer.gov
Transcription factors binding sites prediction software	Transcription factor (TF) binding site prediction is very important in deciphering gene regulation at a transcriptional level. TF binding sites are typically identified by either matching to a consensus sequence or using position-specific scoring matrices (PSSMs). PSSMs can be obtained from resources including the commercial transcription factor database (TRANSFAC) and the open access database JASPAR: Computer methods to locate signals in nucleic acid sequences.^584^ TRANSFAC and its module TRANSCompel: transcriptional gene regulation in eukaryotes.^585^ JASPAR 2016: a major expansion and update of the open-access database of transcription factor binding profiles.^586^ In 2005, Tompa M *et al*^587^ evaluated 13 algorithms designed to identify *cis*-regulatory sites using TF binding sites from TRANSFAC. Their results revealed that the Weeder algorithm performed best: Assessing computational tools for the discovery of transcription factor binding sites.^587^ A set of de novo motif discovery tools, namely rGADEM (R-based genetic algorithm-guided formation of spaced dyads coupled with an expectation-maximization (EM) algorithm for motif discovery), HOMER (hypergeometric optimization of motif enrichment), MEME-ChIP (multiple EM for motif elicitation-chromatin immunoprecipitation), and ChIPMunk (a modification of the classical EM approach), were also evaluated using ChIP-seq data ENCODE. The study showed that rGADEM was the best-performing tool for creating PSSMs from high-throughput ChIP-seq data. FIMO (Find Individual Motif Occurrences) and MCAST (Motif Cluster Alignment and Search Tool) were the best-performing TF binding site prediction tools for scanning PSSMs against DNA: Evaluating tools for transcription factor binding site prediction.^588^	
Tools for neoantigen prediction	Neoantigens are small peptides derived from mutated proteins in cancer cells that can be recognized as foreign by immune cells and trigger an immune response. There are many challenges in computational methods/tools to identify neoantigens and to predict which may serve as optimal targets for the development of immunotherapy approaches: Neoantigens in cancer immunotherapy^589^ Computational genomics tools for dissecting tumour-immune cell interactions.^590^ Applications of immunogenomics to cancer.^490^ MHC binding has been considered a necessary step for neoantigens to be recognized by T cell receptors. The MHC binding prediction methods can be categorized as binding motif-based, position-specific score-based or matrix-based, and machine learning-based, such as artificial neural networks (ANN) or support vector machines. Because of the polymorphic nature of MHC class II molecules and variations in accepted peptide length, the prediction results for MHC class II binding are less accurate than those for MHC class I. Many existing MHC binding peptide and T cell epitope databases could potentially serve as a training data pool to develop prediction models. A good example is the Immune Epitope Database (IEDB), which provides a comprehensive resource for experimental data on antibody and T cell epitopes studied in multiple diseases: SYFPEITHI: database for MHC ligands and peptide motifs.^591^ Profile analysis: detection of distantly related proteins.^592^ Gapped sequence alignment using artificial neural networks: application to the MHC class I system.^593^ NetMHCpan-3.0; improved prediction of binding to MHC class I molecules integrating information from multiple receptor and peptide length datasets.^594^ NetMHCpan, a method for MHC class I binding prediction beyond humans.^595^ Application of support vector machines for T-cell epitopes prediction.^596^ SVMHC: a server for prediction of MHC-binding peptides.^597^ The immune epitope database and analysis resource: from vision to blueprint.^598^ The immune epitope database (IEDB) 3.0.^599^ IEDB: http://tools.iedb.org/main/datasets^600^ Not all MHC binding peptides are immunogenic. Combination approaches have been developed to use additional information (eg, proteasome cleavage) in order to reduce the false positive rate. Since the stability of the peptide–MHC interaction has experimentally been shown to be more strongly correlated to T cell immunogenicity, netMHCstabpan (pan-specific prediction of peptide–MHC class I complex stability) uses a neural network approach based on a data set of stability values calculated for different peptide–MHC class I complexes, rather than their binding affinity values: Pan-specific prediction of peptide-MHC class I complex stability, a correlate of T cell immunogenicity.^601^ Many pipelines have been developed for neoantigen prediction from WES sequencing data via integration of multiple methods. For example, MuPeXI (mutant peptide extractor and informer) is a program to identify tumor-specific peptides from sequencing data and assess their potential to be neoantigens. The peptides are sorted according to a priority score which is intended to roughly predict immunogenicity. A flexible, streamlined computational workflow for identification of personalized Variant Antigens by Cancer Sequencing (pVACSeq) integrates tumor mutation and expression data: MuPeXI: prediction of neo-epitopes from tumor sequencing data.^602^ pVAC-Seq: A genome-guided in silico approach to identifying tumor neoantigens.^603^	IEDB: http://tools.iedb.org/main/datasets
CTRs	USA: https://www.clinicaltrials.gov Europe: https://www.clinicaltrialsregister.eu/	

ANN, artificial neural networks; CIDC, Cancer Immunologic Data Commons; CIMAC, Cancer Immune Monitoring and Analysis Centers; CTR, clinical trial registry; EM, expectation maximization; FDA, Food and Drug Administration; FIMO, Find Individual Motif Occurrences; HOMER, hypergeometric optimization of motif enrichment; IEDB, Immune Epitope Database; MCAST, Motif Cluster Alignment and Search Tool; MHC, major histocompatibility complex; MuPeXI, mutant peptide extractor and informer; NCI, National Cancer Institute; NIAID, National Institute of Allergy and Infectious Diseases; PACT, Partnership for Accelerating Cancer Therapies; PSSM, position-specific scoring matrix; pVACSeq, peronsalized Variant Antigens by Cancer Sequencing; rGADEM, R-based genetic algorithm-guided formation of spaced dyads coupled with an EM algorithm for motif discovery; TF, transcription factor; WES, whole exome sequencing.
